# Do foraging methods in winter affect morphology during growth in juvenile snow geese?

**DOI:** 10.1002/ece3.2481

**Published:** 2016-10-05

**Authors:** Jón Einar Jónsson, Alan D. Afton

**Affiliations:** ^1^ Research Centre at Snæfellsnes University of Iceland Stykkishólmur Iceland; ^2^ U.S. Geological Survey Louisiana Cooperative Fish and Wildlife Research Unit Louisiana State University Baton Rouge LA USA

**Keywords:** bill size, body size, foraging exertion, habitat selection, introgressive hybridization, morphotypes

## Abstract

Physical exertion during growth can affect ultimate size and density of skeletal structures. Such changes from different exercise regimes may explain morphological differences between groups, such as those exhibited by lesser snow geese (*Chen caerulescens caerulescens*; hereafter snow geese) foraging in southwest Louisiana. In rice‐prairie habitats (hereafter rice‐prairies), snow geese bite off or graze aboveground vegetation, whereas they dig or grub for subterranean plant parts in adjacent coastal marshes. Grubbing involves considerably more muscular exertion than does grazing. Thus, we hypothesized that rates of bone formation and growth would be lower for juveniles wintering in rice‐prairies than those in coastal marshes, resulting in smaller bill and skull features at adulthood. First, we tested this exertion hypothesis by measuring bills, skulls, and associated musculature from arrival to departure (November–February) in both habitats in southwest Louisiana, using both banded birds and collected specimens. Second, we used the morphological data to test an alternative hypothesis, which states that smaller bill dimensions in rice‐prairies evolved because of hybridization with Ross's geese (*C. rossii*). Under the exertion hypothesis, we predicted that bill and skull bones of juveniles would grow at different rates between habitats. However, we found that bill and skull bones of juveniles grew similarly between habitats, thus failing to support the exertion hypothesis. Morphometrics were more likely to differ by sex or change with sampling date than to differ by habitat. We predicted that significant, consistent skewness toward smaller birds could indicate hybridization with Ross's geese, but no skewness was observed in our morphological data, which fails to support the hybridization hypothesis. Further research is needed to clarify whether snow geese wintering in Louisiana represent a single polymorphic population that segregates into individually preferred habitats, which we believe at present to be more likely as an explanation than two ecologically and spatially distinct morphotypes.

## 1 Introduction

Niche expansion is an adaptation to changes in food availability that often leads to divergent selection toward the use of alternative resources (Benkman, 2003; Grant & Grant, 1989). Morphological changes may occur, in turn, resulting in ecologically segregated morphs and eventually leading even to different species (Kleindorfer, Chapman, Winkler, & Sulloway, 2006; Scott, Clegg, Blomberg, Kikkawa, & Owens, 2003). Bill morphology often responds to changing selective pressures resulting from changes in the diet or the characteristics of the niche (Grant & Grant, 2002; Grenier & Greenberg, 2005; Scott et al., 2003), although genetics of growth or body size can change with environmental conditions (Larsson, Rattiste, & Lilleleht, 1997; Larsson, van der Jeug, van der Veen, & Forslund, 1998).

**Figure 1:**
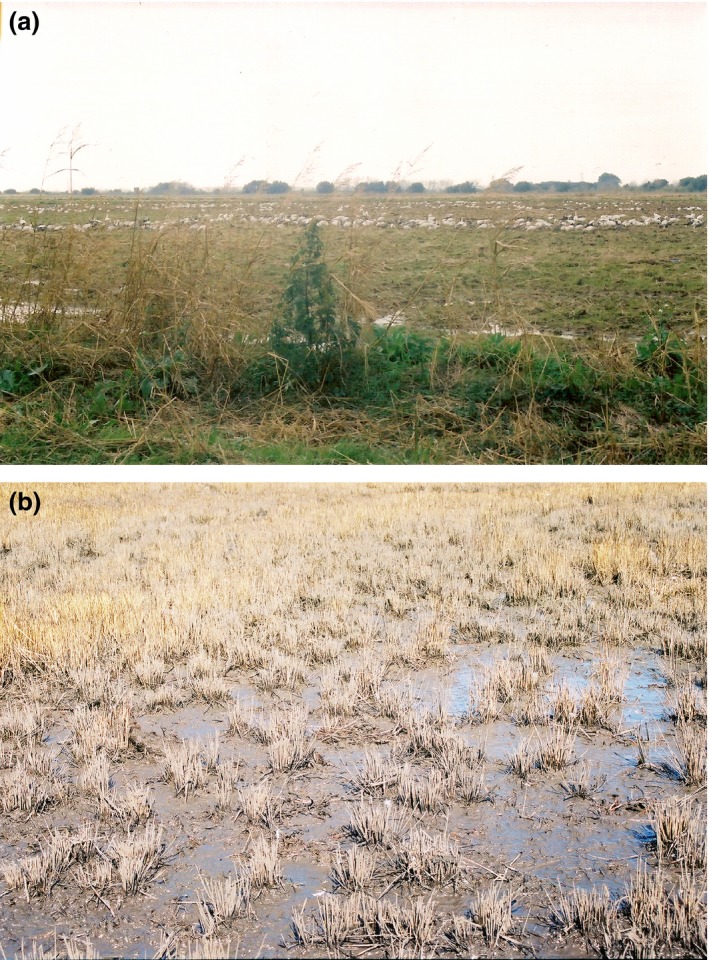
(a) Rice‐prairie near Sweet Lake, Calcasieu Parish, Louisiana. Lesser snow geese (*Chen caerulescens caerulescens*) are grazing on aboveground vegetation in the background (photograph by Jón Einar Jónsson). (b) Coastal marsh, State Wildlife Refuge, Cameron Parish, Louisiana. The patches of mud in the foreground were created by grubbing lesser snow geese. The area in the background was left relatively intact by the snow geese (photograph by Jón Einar Jónsson)

**Figure 2:**
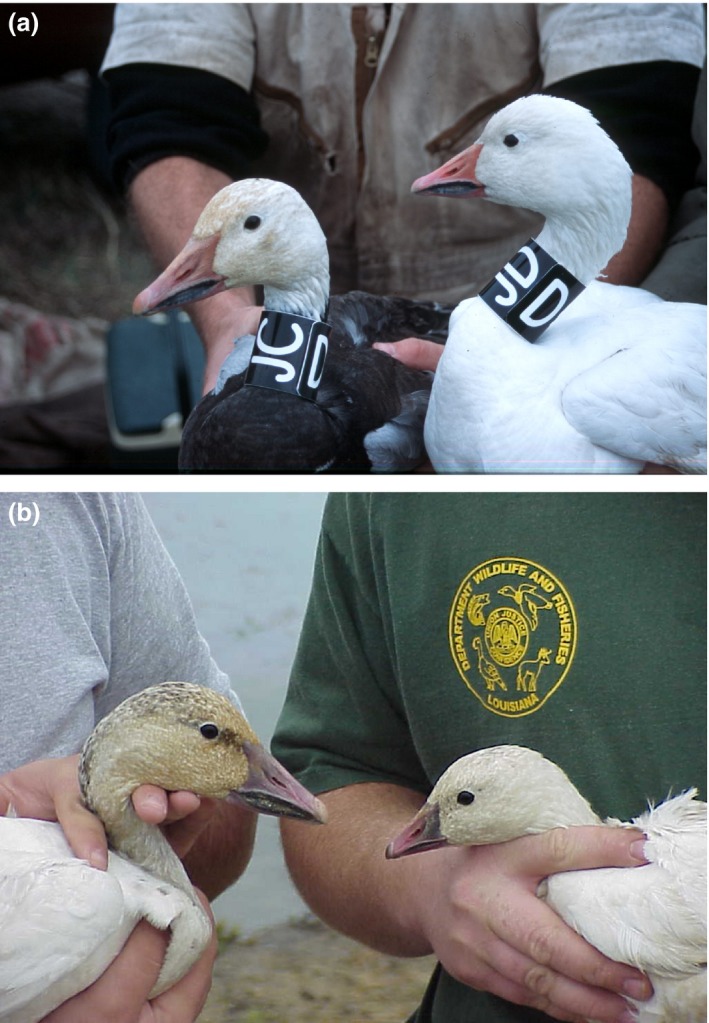
(a) Lesser snow geese (*Chen caerulescens caerulescens*) banded at Cameron Prairie National Wildlife Refuge, Calcasieu Parish, Louisiana, in November 2001. The snow goose (blue color phase) on the left is representative of snow geese from a coastal‐marsh habitat, whereas the snow goose (white color phase) on the right is representative of snow geese from a rice‐prairie habitat (photograph by Jón Einar Jónsson). (b) A comparison of a lesser snow goose (*Chen caerulescens caerulescens*) to the left and a Ross's goose (*C. rossii*) to the right banded at Rockefeller State Wildlife Refuge, Cameron Parish, Louisiana, in January 2002. Note that Ross's geese are two‐thirds the size of lesser snow geese, with a shorter neck and a smaller and more rounded head, their bill is “higher at the base, tapers steeply to a rounded tip, has a slight arch in the tomium of maxilla and mandible, but lacks the prominent dark ‘grinning’ or ‘smile’ patch characteristic of lesser snow geese” (Jónsson et al., 2013) (photograph by Rockefeller SWR staff)

Lesser snow geese (*Chen caerulescens caerulescens*; hereafter snow geese) use rice‐prairie (Figure 1a) and coastal‐marsh habitats (Figure 1b) in southwest Louisiana during winter (Alisauskas, 1998; Alisauskas, Ankney, & Klaas, 1988; Jónsson & Afton, 2006, 2015; Jónsson, Frederiksen, & Afton, 2014). Snow geese in coastal marshes have larger bodies and proportionally thicker bills, longer skulls, and longer culmens than do geese in rice‐prairies (Alisauskas, 1998; Jónsson, 2005; Figure 2a). Among geese, larger bills are better suited for digging up belowground plant parts and for dealing with tough food items (Alisauskas, 1998; Black & Owen, 1990; Owen, 1980). In coastal marshes, snow geese forage primarily by digging, or grubbing, for belowground parts of vegetation, such as tubers of the Olney bulrush (*Scirpus olneyi)* and saltmarsh bulrush (*Scirpus robustus*) and rhizomes of marshhay cordgrass (*Spartina patens)* and saltgrass (*Distichlis spicata*) (Alisauskas et al., 1988). In rice‐prairies, snow geese mostly graze on agricultural plants and consume aboveground vegetation, which are mechanically easier to gather than belowground plant parts in coastal marshes (Alisauskas, 1998; Alisauskas et al., 1988; Batt, 1997).

Bill size is positively related to the rate of food intake, and differences in bill size may cause biting mechanisms to vary among individuals because the angle of the gape increases with the length of the bill (Bock, 1972; Cope, Loonen, Rowcliffe, & Pettifor, 2005; Durant, Fritz, Blais, & Duncan, 2003). Thus, a tall bill is beneficial for species that forage on large or tough food items (Grant & Grant, 1989), such as rhizomes and tubers. Furthermore, foraging in coastal marshes is more energetically costly and time‐consuming for snow geese than foraging in rice‐prairies because (1) grubbing requires approximately 1.5 times more energy and greater activity than does grazing (Bolen & Rylander, 1978; Gauthier, Bédard, & Bédard, 1984); and (2) adult snow geese in coastal marshes spend 12% more time foraging than adult snow geese in rice‐prairies (Jónsson & Afton, 2006). Furthermore, foraging methods differ in the mechanical loadings exerted on neck muscles (van der Leeuw, Bout, & Zweers, 2001) as well as those acting on muscles, bones, and tissues of the skull, bill, and head.

The occurrence of two ecological morphs among wintering snow geese may be the result of different mechanisms that can be explained by at least four competing hypotheses (Table 1). Geese with smaller bills could be selected against in the coastal‐marsh habitat, whereas those with larger bills may be effective for both grubbing and grazing and, hence, are found in both coastal marshes and rice‐prairies (phenotypic selection hypothesis; Alisauskas, 1998; Table 1). Within a species, larger‐billed individuals can feed on larger as well as small food items, whereas smaller‐billed individuals generally are restricted to feeding on smaller food items (Lederer, 1975; Smith, 1990; Willson, 1972). Alternatively, the occurrence of two ecological morphs may be the result of larger‐billed individuals selecting the coastal marshes, while smaller‐billed individuals restrict themselves to rice‐prairies (habitat selection hypothesis; Alisauskas, 1998; Table 1).

**Table 1:** Hypotheses and their predictions for explaining morphological segregation of juvenile snow geese into two habitats (rice‐prairies and coastal marshes) in southwest Louisiana in winters 2001–2004. Note that hypotheses are not necessarily mutually exclusive and only the introgressive hybridization and exertion hypotheses were tested in the study

Hypothesis	Consequences for rice‐prairie snow geese	Consequences for coastal‐marsh snow geese	Prediction	Source
Phenotypic selection—natural selection drives the segregation	No selection against smaller body size or bill size within the habitat	Small body size or bill size is selected against within the habitat	Coastal‐marsh snow geese are larger than rice snow geese	Alisauskas (1998)
Habitat selection—habitat choices by individuals drive the segregation	Individuals with small body size or bill size select this habitat	Individuals with small body size or bill size avoid this habitat	Coastal‐marsh snow geese are larger than rice snow geese	Alisauskas (1998)
Introgressive hybridization—segregation is due to hybridization with the smaller, closely related Ross's geese	Genetic material for smaller body size or bill size is mixed into the population in this habitat (Ross's geese are present)	Genetic material for smaller body size or bill size is not available in this habitat (Ross's geese are absent)	Distribution(s) show skewness or bimodality within the rice‐prairies only	Alisauskas (1998)
Exertion—physical exertion varies between habitats and exertion during growth results in different‐sized adult populations	This habitat requires little physical exertion during feeding	This habitat demands considerable physical exertion during feeding	Coastal‐marsh juveniles show a greater increase in morphometric indices from early to late winter than those in rice‐prairies	Gauthier, Bedard and Bedard (1994), Biewener & Bertram (1994)

A further complication is that the smaller sympatric Ross's geese (*Chen rossii*; Figure 2b) commonly form mixed flocks with snow geese in rice‐prairies (Jónsson & Afton, 2008 and Jónsson & Afton, 2009). Ross's geese hybridize with snow geese (Jónsson, Ryder, & Alisauskas, 2013; Weckstein, Afton, Zink, & Alisauskas, 2002), and Alisauskas (1998) hypothesized that the smaller bodies and bill sizes among the snow geese in the rice‐prairies could be explained by introgressive hybridization (see also Rheindt & Edwards, 2011) between Ross's geese and snow geese. Alisauskas (1998) termed this the “introgressive hybridization hypothesis” (Table 1). Hybridization between species can be detected in morphological data by skewed distributions of measurements (Grant & Grant, 2008). We predicted that such skewness had to occur within rice‐prairies but not necessarily within the coastal marshes, because Ross's geese rarely use coastal marshes (Alisauskas, 1998; Jónsson, 2005; Jónsson & Afton, 2009).

Environmental conditions during growth, such as condition of food items eaten, may affect adult body size (Larsson & Forslund, 1991; Larsson et al., 1997). Thus, it may not be possible to infer selection on morphometrics of adults without considering environmental conditions during growth (Larsson et al., 1998). Geese do not reach full growth until after they reach at least 1 year (2 years as indicated by birds caught on breeding grounds) of age and their growth rates affect final adult size (Cooch, Lank, Dzubin, Rockwell, & Cooke, 1991; Davies, Rockwell, & Cooke, 1988; Larsson & Forslund, 1991), and thus, the two ecological morphs could be the result of differences in the regime of physical exertion that the snow geese undergo during their first winter before adulthood, assuming that grubbing for coastal‐marsh food is physically more demanding than grazing for rice‐prairie food (hereafter termed “exertion hypothesis”; Table 1).

**Figure 3:**
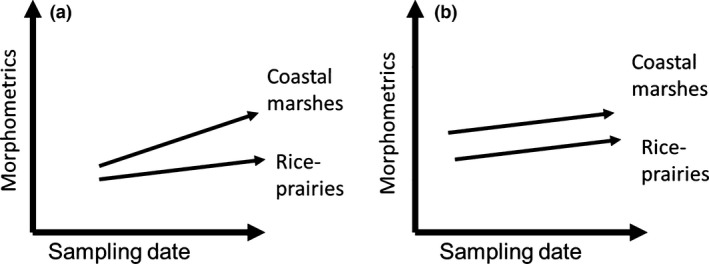
Diagram illustrating the predicted relationships between morphometric variables of growing lesser now geese (*Chen caerulescens caerulescens*) in coastal marshes and rice‐prairies in relation to sampling date (20 November to 17 February). (a) If the morphometric variables of coastal‐marsh snow geese grow faster over the winter months than do those of rice‐prairie snow geese due to the greater exertion needed to forage for coastal‐marsh food, the exertion hypothesis would be supported. (b) If the morphometric variables of the marsh and rice‐prairie snow geese grow at the same rate, the exertion hypothesis is not supported

Physical exertion (muscular exercise or load bearing) during growth can contribute significantly to the buildup of bone mass, its mineral content, proportions, weight, width, and length that is reached in adulthood (Auerbach & Raxter, 2008; Bailey, Faulkner, & McKay, 1996; Biewener & Bertram, 1994; Judex & Zernicke, 2000), although different bones or sites within bones may differ in their responses to the same stimuli (Regmi et al., 2015; Wallace et al., 2015) and experimentally induced high levels of physical activity may actually lead to reduced bone growth (Foutz, Griffin, Halper, & Rowland, 2007). We are not aware of any controlled experiments that specifically measured effects of mechanical loadings on goose skulls or bills, or any that showed that muscle changes affect these bones during growth. However, mechanical loadings are important vectors affecting bone shape and joint integrity in developing embryos (Nowlan, Sharpe, Roddy, Prendergast, & Murphy, 2010). Moreover, the avian bill shows great plasticity in general, and the skull has muscular connections with the neck muscles. Thus, these bones plausibly respond to some of the same mechanical loadings caused by the neck muscles, and these loadings differ between foraging methods (van der Leeuw et al., 2001). Differences in bill thickness, culmen length, head width, and head height of adult snow geese in rice‐prairies and coastal marshes reported by Alisauskas (1998) therefore may have resulted from different intensities of physical exertion while foraging during the first winter, while juvenile snow geese are still growing. Juvenile snow geese feeding in rice‐prairies probably require less muscular exertion via feeding than those feeding in coastal marshes. Thus, bone densities and growth rates of juveniles feeding in rice‐prairies may be lower than those of those feeding in coastal marshes, ultimately resulting in smaller bills, skulls, and culmens in adults. According to the exertion hypothesis, juveniles feeding in coastal marshes are predicted to grow faster and show a greater increase in morphometric indices from early to late winter than those feeding in rice‐prairies (Figure 3).

We examined these four competing hypotheses by comparing the sizes of several characters related to the feeding behavior of juvenile geese in rice‐prairies and coastal marshes throughout the wintering season, to determine their respective rates of growth. Specifically, we used morphological data to test the exertion and introgressive hybridization hypotheses, and also discuss our findings in relation to the phenotypic selection and habitat selection hypotheses.

## 2 Methods

### 2.1 Study area

Our study area (10,764 km^2^) in southwest Louisiana was bordered by Sabine National Wildlife Refuge (29°53′N, 93°23′W) on the west; Lake Charles and Highway 383 on the northwest; Highway 190 on the north; Highway 387 and Interstate 10 on the northeast; Highway 35 on the east; and the Gulf Coast on the south (Jónsson et al., 2014). The ecology of rice‐prairies and coastal marshes was described in detail by Alisauskas (1988), Alisauskas et al. (1988), Bateman, Joanen, and Stutzenbaker (1988), and Jónsson (2005).

The Intracoastal Canal generally separates coastal marshes and rice‐prairies in southwest Louisiana (Bateman et al., 1988). Coastal marshes are comprised of fresh, intermediate, brackish or saline wetlands, but fresh and intermediate wetlands are not used frequently by snow geese. The coastal brackish and saline wetlands in coastal marshes are separated by about 32 km from the rice‐prairies, which also are used by snow geese (Bateman et al., 1988). Rice‐prairies are former tallgrass prairies that have been extensively cultivated, mostly for rice, but also as pastures for cattle (Alisauskas, 1988; Alisauskas et al., 1988; Bateman et al., 1988). Snow geese wintered exclusively in coastal marshes until the 1940s, but they began using agricultural lands 20–30 miles inland within the last 80 years, particularly those planted with rice *Oryza sativa* (hereafter rice‐prairies) (Bateman et al., 1988).

### 2.2 Banding and collections of juvenile snow geese

We used two methods to obtain snow geese for measurements. First, we caught a total of 106 juvenile snow geese using rocket‐nets (Dill & Thornsberry, 1950) and then banded, and released them (Jónsson, 2005; Jónsson et al., 2014): (1) 21 females and 22 males in rice‐prairie habitats at Cameron Prairie National Wildlife Refuge and Oak Island (30°00′N, 92°04′W), 10 miles south of the town of Lake Arthur in Louisiana; and (2) 33 females and 30 males in coastal marshes at Rockefeller State Wildlife Refuge (29˚40′N, 92˚55′W) and Sabine National Wildlife Refuge (29°53′N, 93°23′W). Snow Geese were banded in rice‐prairies from 20 November to 10 February and from 17 December to 20 January in coastal marshes. Banding efforts began in November 2001 and ended in January 2004 (Jónsson et al., 2014). Hereafter, the measured birds are termed “coastal‐marsh” and “rice‐prairie,” according to their banding sites (Jónsson et al., 2014); these terms are not to be confused with the terms for the two separate morphs or populations.

Second, we collected a total of 71 juvenile snow goose specimens from 20 November to 17 February in the winters of 2001–2002, 2002–2003, and 2003–2004, using .22 caliber rifles and 12‐gauge shotguns: 16 females and 19 males in rice‐prairies of Sweet Lake (8–16 km north of Cameron Prairie National Wildlife Refuge) or within 24 km west, or south, of the town of Lake Arthur at Oak Island (30°00′N, 92°04′W) or Thornwell (30°10′N, 92°80′W), including Oak Island; and (2) 21 females and 15 males in coastal marshes at Rockefeller State Wildlife Refuge (29°40′N, 92°55′W). Collected specimens were individually double‐bagged and frozen, and subsequently stored in a walk‐in freezer at Louisiana State University. Hereafter, specimens are termed “coastal‐marsh specimens” and “rice‐prairie specimens,” according to their collection sites; these terms are not to be confused with the terms for the two separate morphs or populations.

Banded birds and collected specimens were sexed by cloacal examination (see Hochbaum, 1942) and aged by plumage color as either adult (after‐hatch‐year and older) or juveniles (hatch‐year) (see Baldassarre, 2014). Juvenile snow geese were banded or collected on different dates throughout the winter, and we used sampling date (20 November to 17 February) to index the juvenile growth period. We measured different individuals at different times within the wintering period but did not measure growth rates within individuals. Thus, our study assumed that there were no differential migrations by bill size or body size, in or out of our study area, within our sampling period (November–February). Sampling date was included as a covariate in all our analyses; 20 November was designated as sampling date 1 and 17 February as sampling date 90.

We caught, banded, and collected geese under the U.S. Fish and Wildlife Service scientific collection permit MB048372‐0, Louisiana Department of Wildlife and Fisheries scientific collection permit LNHP‐01‐052, banding permit 08810‐A from the U.S. Geological Survey Bird Banding Lab, Cameron Prairie National Wildlife Refuge special permit use permit 43612‐03004, Sabine National Wildlife Refuge special use permit 43640‐02028, and Louisiana State University Agricultural Center Institutional Animal Care and Use Committee (LSU AgCenter IACUC) permit number A01‐09.

### 2.3 External measurements

**Figure 4:**
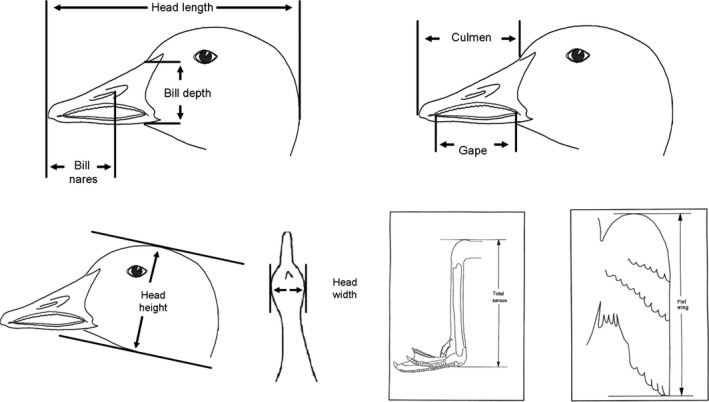
External measurements taken from banded birds and collected specimens of lesser snow geese (*Chen caerulescens caerulescens*). A total of 177 juveniles were banded or collected, and subsequently measured in southwest Louisiana in winters 2001–2002, 2002–2003, and 2003–2004. Drawn after and modified from Dzubin and Cooch (1992)

For both banded birds and collected specimens (*n* = 177), we measured the following (Figure 4) with calipers (±0.1 mm): (1) head length from the upper bill tip (distal part of egg tooth) to the nape; (2) bill nares, that is, diagonal length of the upper bill measured from the rostral edge of the nostril); (3) bill thickness (upper bill) from the posterior lateral extension to the base of the commissural point); (4) culmen length; (5) gape length; (6) head width, that is, the distance between the lateral sides of the head; (7) head height, that is, the distance between the dorsal and ventral sides of the head; (8) total tarsus, that is, the diagonal length from the palpable medial‐most condyle of the tarsus where it articulates with the mid‐phalange (toe), to the palpable rounded exterior portion of the distal condyles of the tibia; and (9) flat wing on a wing board (see also Alisauskas, 1988, 1998; Dzubin & Cooch, 1992). The terminology for the external measurements follows that of Dzubin and Cooch (1992), except for head width and head height, which follows that of Alisauskas (1998). Note that the skin was never removed, and thus, we use “head” instead of “skull” when naming head height and head width, which are synonymous with skull height and skull width, respectively (Alisauskas, 1998).

### 2.4 Muscle measurements

Collected specimens (*n* = 71) were thawed at room temperature for 24–48 hr prior to measuring and dissection, following the methods of Alisauskas (1988). We measured or weighed (1) the paired dorsal neck muscles as a single unit by excising the dorsal neck muscles between the third vertebra and the occiput of the skull after penetrating the surrounding skin and fascia with a pair of forceps; (2) the paired external adductor mandibulae muscles of the jaw by cutting them off their attachment sites after penetrating the surrounding fascia; and (3) the paired depressor mandibulae muscle of the jaw excising them from their attachment sites after removing surrounding fascia. These are the muscles involved in the grabbing and pulling at food items (leaves, tubers, rhizomes) while foraging (grazing and grubbing) and, thus, should respond differently to different foraging methods between habitats.

All muscles were weighed with a digital scale to ±0.1 g and measured immediately after excision. The diameter of neck muscles and jaw muscles were measured at three locations, namely within 2 mm from each attachment and at mid‐length, and the average from these three measurements was used for analysis (hereafter called “muscle diameter” at ±0.1 mm). The shape of the excised depressor mandibulae muscles of the jaw was too irregular for measuring the muscle diameter. The muscle diameters of paired muscles were averaged, and the weights of all paired muscles (except the unpaired dorsal neck muscles) were combined (hereafter called “muscle weight” at ±0.1 g) for the subsequent statistical analysis.

### 2.5 Statistical analysis

In this study, we intended to solely use an information theory approach to model selection (Anderson, 2008) to test our study hypotheses. However, we employ hypothesis testing when particular statistics are not amenable to model selection approaches, that is, for testing for skewness and bimodality. Our analyses of external measurements were conducted on all such measurements (*n* = 177) from banded birds (*n* = 106) and collected specimens (*n* = 71) combined but stratified by sex, habitat, and method.

### 2.6 Adjusting muscle measurements for individual body size

Absolute measurements generally are less precise in populations with individually variable body size, and it is standard practice to standardize measurements to some value representative of body size (Relyea, 2004). For the muscular measurements, we analyzed individual variation in body size. Here, a principal components analysis (PCA, PROC PRINCOMP of SAS Institute Inc., Cary, Indiana) was based on external measurements from collected specimens only (*n* = 71). (Conversely, when we later analyzed the external measurements after grouping them into fewer dimensions by PCA, we used a PCA on all measured birds (banded + collected), prior to analyzing differences by sex, method, habitat, and sampling date with linear mixed models.) In the body size‐adjustment PCA, the first principal score (PC1) had meaningful loadings for all nine external measurements, as previously reported by Alisauskas (1998). PC1 (which later is included in model selections for external measurements) explained 53.0% of the overall variation and, thus, is a useful index of body size. We proceeded by regressing PC1 on each measurement (nine muscle measurements, yielding nine regressions) to obtain the individual's residual value. We then added the residual value to the overall mean measurement to get those particular individuals size‐adjusted value (mean + residual).

### 2.7 The test of the exertion hypothesis

Body size measurements of geese are not independent of each other, and over half the variation in external measurements can be explained by body size, rather than by body shape (Alisauskas, 1998; Jónsson, 2005; Jónsson et al., 2014). Thus, we reduced dimensions among our response variables by performing separate PCA on the correlation matrices for the following: (1) the nine external measurements; and (2) the nine muscle measurements. These PCA created new linear combinations from the measurements, that is, nine principal scores in each analysis which are completely orthogonal to one another; and thus, independent metrics of size and various shapes based on the measurements. We inspected principal scores for subsequent analyses based on linear combinations of parameters of interest, which were bill dimensions (bill nares, bill thickness, culmen length, gape length), but also the eigenvalues and cumulative variation explained by each principal score. We followed Alisauskas (1998) for interpretation of eigenvectors (loadings) of principal scores and Hamel and Côté (2008) for interpretation of PC score eigenvalues.

We ran the PCAs to reduce dimensions but also because we knew a priori that the PCA would segregate variation due to body size by linearly combining such variation into the first principal score, which previously has explained 49%–55% of the overall variation in adult snow geese wintering in southwest Louisiana (PC1; Alisauskas, 1998; Jónsson et al., 2014). Furthermore, PC1 in adult snow geese can differ by sex and habitat, at least in some years (Alisauskas, 1998; Jónsson, 2005).

We then proceeded to use PCA scores with eigenvalues ≥1.0 for analyses. We also kept PC scores with lower eigenvalues if their loadings were relevant to our study hypotheses by containing bill dimensions or neck, skull, or bill musculature measurements. These two types of relevant PC scores were then used as response variables in linear mixed models with three fixed effects: method (collected specimens or banded live birds), sex (male or female), and habitat (rice‐prairies or coastal marshes). Furthermore, sampling date was included as a covariate, which by definition is a random effect because our collected specimens represented a larger population. Habitat was important for our research hypotheses. Sexual size dimorphism is present in snow geese, both among adults and at the gosling stage (Alisauskas, 1998; Aubry et al., 2013 Cooch, Lank, & Cooke, 1996 and; Cooch, Lank, Robertson, & Cooke, 1997; Cooke, Rockwell, & Lank, 1995; Jónsson, 2005). Although males are only 2%–6% larger than females at all ages (Cooch et al., 1997), we included sex in our analysis to control for this variation. Method was included to account for potential variation in measurements between banded birds and collected, frozen specimens because measurements from the latter method may be affected from freezer shrinkage after being stored in a freezer for a few months prior to the dissections (Bjordal, 1983).

Following Anderson (2008), we included model parameters that were of biological interest with respect to our research questions and tests of hypotheses. We did not include any interactions involving method because: (1) JEJ measured all collected specimens in the laboratory; and (2) in the field, we used standardized morphometrics that have good repeatability and assume that any variance among individuals that performed these measurements would be small and unimportant in our analysis. Furthermore, we see no reason why the effects of method should depend on habitat or sex, or vice versa. We expected a priori that measurements would differ between the sexes, but saw no reason why sex effects should depend on habitat or sampling dates, or vice versa. We included the habitat × sampling date interaction as a random effect, because it was the statistical test of the exertion hypothesis; that is, measurements should differ by sampling date but only dependent on habitat under this hypothesis (Figure 3).

The exertion hypothesis is specific to skull dimensions (head width and head height) and bill dimensions (culmen length, bill nares, bill thickness, and gape length). The PCA created new linear combinations of the morphometric measurements. We inspected PC2–PC9 for linear combinations of bill dimensions and PC2–PC5 for muscle measurements, whereas general variation in body size was contained in PC1. The exertion hypothesis does not state that rice‐prairie snow geese grow to smaller adults, but specifically that their bills, relative to the rest of the body, grow to be smaller than those of coastal‐marsh snow geese. While we see no reason why PC1 would respond to variable exercise between habitats, should it exist, we present PC1 and kept it for linear mixed model analysis to evaluate with information theory the relative importance of habitat and sex in snow geese, and to examine whether PC1 would behave similarly for external measurements and muscle measurements.

We used AIC model selection, using the AICcmodavg package in R (Mazerolle, 2015) to compare linear mixed models for important PC scores, pertaining to our study hypotheses, from both external measurements and muscle measurements. Our model building followed these steps: (1) We ran the intercept‐only models (also termed null models); (2) we ran the fixed‐effects model sex + habitat + method and all nested models, that is, sex + habitat, sex + method, habitat + method, and single‐effects models for each fixed effect; and (3) we only added the random effects, sampling date and sampling date × habitat, to the intercept‐only models (creating the random‐effects models) or to the all fixed‐effects models. Method was never included in linear models for muscle measurements because all birds were collected specimens in that dataset. We paid particular attention to possible pretender variables (Anderson, 2008; Arnold, 2010); that is, candidate models which are within ΔAIC ≤ 2.0 of the top‐ranked model differ from the top‐ranked model by an additional 1–2 variables, yet their log‐likelihood values are almost the same as those of the top‐ranked model. Such observations suggest that the model with the additional variable really adds very little information to the top‐ranked model. We used cumulative weights (sums of Wi) to evaluate differences between best models and probable pretender variables (Burnham and Anderson 2002).

### 2.8 Test of introgressive hybridization hypothesis

We inspected the distributions for each individual external measurement for skewness and bimodality. We first stratified these distributions by method (banded or collected) and sex (male or female) and then proceeded with Student's *t*‐test between the mean and median to test for skewness in the data (Sokal & Rohlf, 1995). For each of the nine external measurements, we used false discovery rates (FDR) within each set of eight tests (combinations of sex, habitat, and method) and used *p*‐values from all the comparisons to calculate FDR threshold α‐levels (α < .05), to evaluate against each *p*‐value of from Student's *t*‐tests. Here, we present findings from the classical one‐stage method for FDR (Pike, 2011).

Significant, consistent skewness toward smaller birds could indicate hybridization with Ross's geese, particularly if such skewness were to occur within rice‐prairies but not coastal marshes. We also visually inspected these distributions for potential bimodality, which would be another sign of potential hybridization.

## 3 Results

### 3.1 Test of the exertion hypothesis

#### 3.1.1 Three principal scores for external measurements

The first two principal scores (PC1 and PC2), which explained 53% and 13% of the cumulative variation, respectively, were the only PC scores with eigenvalues ≥ 1 and cumulatively explained 66% of the variation in our data (Table 2). The eigenvectors of PC1 had similar, all positive, loadings indicating, as we expected a priori, that PC1 contained the variation for body size (Table 2). The eigenvectors of PC2 were comprised of negative loadings for many measurements pertaining to the bill, that is, culmen length, bill nares, and gape length, and positive loadings for measurements of head width, head height, and wing length (Table 2). This indicates an inverse relationship of the bill measurements relative to the skull; the positive values may indicate that PC2 represents the bill dimensions relative to the rest of the body. PC2 included all bill dimensions except bill thickness, which was represented by PC3, which explained 7% of the overall variation and had a single, high loading (0.70) for bill thickness (Table 2); thus, PC3 also was analyzed with a linear mixed model testing the exertion hypothesis. Other PC scores (PC4–PC9) had eigenvalues ≤ 0.60 and each explained ≤6.7% of the overall variation, and thus were not considered further.

**Table 2:** Eigenvectors (eigenvalues) from a principal components analysis of morphological measurements of 177 juvenile lesser snow geese banded or collected in southwest Louisiana in winters 2001/02, 2002/03 and 2003/04. Numbers in bold correspond to variables that covaried the strongest with each PC score (i.e., had the highest loadings)

	PC1 (4.8)	PC2 (1.2)	PC3 (0.7)
Head length	**0.42**	−0.14	−0.10
Bill nares	**0.28**	**−0.53**	−0.13
Bill thickness	**0.31**	0.16	**0.70**
Culmen length	**0.35**	**−0.35**	−0.08
Gape length	**0.34**	**−0.35**	0.19
Head width	**0.29**	**0.39**	−0.49
Head height	**0.34**	**0.37**	−0.09
Total tarsus	**0.34**	0.15	−0.29
Wing length	**0.31**	**0.35**	0.32
% variance explained	53	13	7

#### 3.1.2 Three principal scores for muscle measurements

The first three principal scores (PC1, PC2, and PC3 explained 60%, 15%, and 13% of the cumulative variation, respectively) cumulatively explained 88% of the variation in our data (Table 3). The first PC score was the only PC score with eigenvalue ≥ 1, but PC2 and PC3 were also kept for analyses as they referred to diameter of neck and skull muscles (Table 3) and, thus, of potential interest regarding the exertion hypothesis. Other PC scores (PC4–PC5) had eigenvalues ≤ 0.33 and each explained ≤6.6% of the overall variation, and thus were not considered further.

**Table 3:** Eigenvectors (eigenvalues) from a principal components analysis of muscle measurements of 71 juvenile lesser snow geese specimens, collected in southwest Louisiana in winters 2001/02, 2002/03, and 2003/04. Numbers in bold correspond to variables that covaried the strongest with each PC score (i.e., had the highest loadings)

	PC1 (3.0)	PC2 (0.8)	PC3 (0.7)
Total neck muscle mass	**0.48**	0.02	−0.52
Neck muscle diameter	**0.43**	**0.63**	−0.31
Skull muscle mass	**0.46**	−0.47	0.27
Skull muscle diameter	**0.40**	0.39	**0.74**
Jaw muscle mass	**0.46**	−0.48	−0.09
% variance explained	60	15	13

The eigenvectors of PC1 had similar, all positive, loadings for all variables, indicating that PC1 served as an overall muscle size index (Table 3). The eigenvectors of PC2 and PC3 had single high loadings that represented diameters of neck and skull muscles, respectively (Table 3).

#### 3.1.3 Linear mixed models on PC scores for external measurements

**Figure 5:**
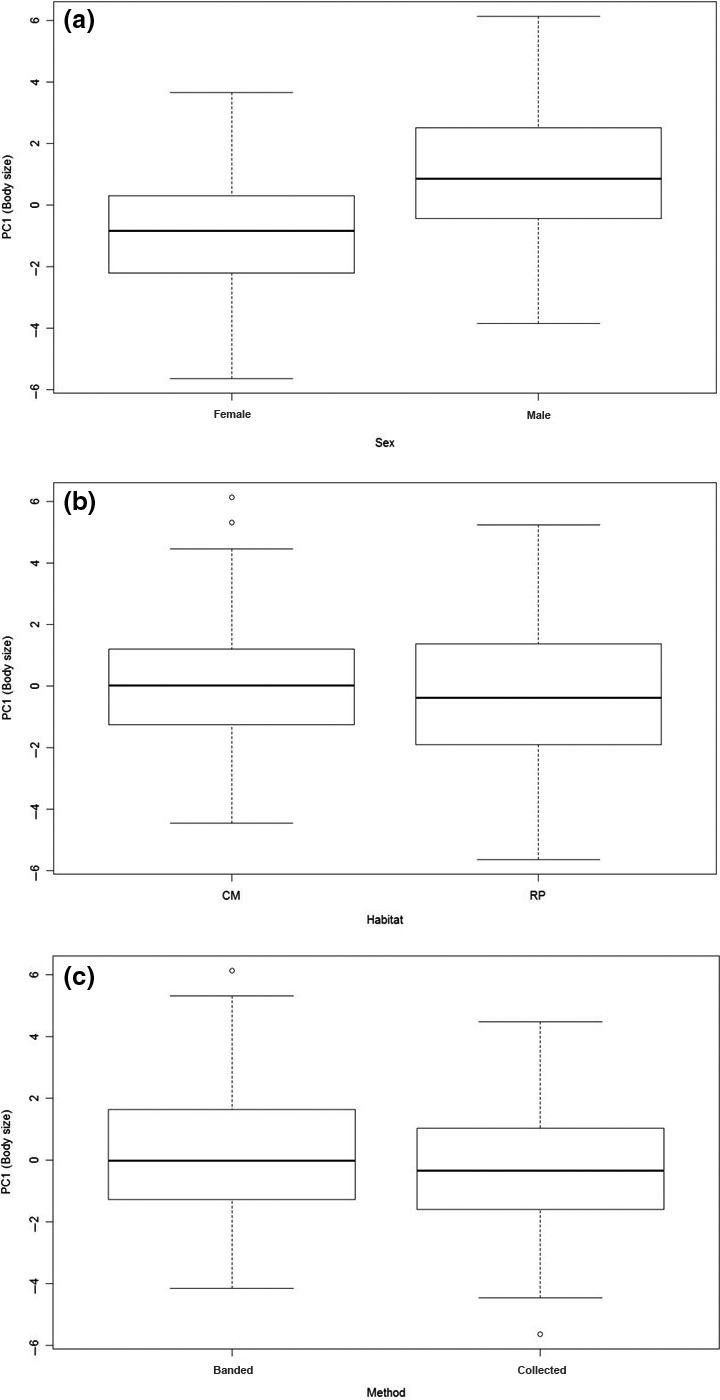
Tukey boxplots (the length of the box is the interquartile range, whiskers are drawn to the largest observations within 1.5 interquartile lengths from the top and bottom) of the differences in body size (PC1) between the sexes (a), habitats (b) (CM, coastal marshes and RP, rice‐prairies), and methods (c), based on external measurements of juvenile snow goose (*Chen caerulescens caerulescens*) specimens (*n* = 171) banded or collected in southwest Louisiana during winters 2001–2004. Males were structurally larger although there is overlap in body size between sexes

For PC1 (which represented body size), the effects of sex were the most important, while there was less support for other variables. Sex, habitat, and method had cumulative weights of 1.00, 0.56, and 0.55, respectively, indicating that sex was the most important among the three fixed effects. However, the top‐ranked model also included habitat and method in addition to sex (Table 4; Appendix S1 for full model selection tables), although adding habitat and method to sex only changed LogL by 1.3 and adding both changed LogL by 2.4. Males were on average structurally larger than females (Figure 5a), whereas body size was similar between habitats but more variable in the rice‐prairie habitat (Figure 5b). Collected specimens were marginally smaller than banded birds but with great overlap between methods (Figure 5c).

**Table 4:** Linear mixed models testing effects of sex, habitat, method, sampling date, and habitat × sampling date on snow goose morphometrics. Dependent variables were each of three principal components (PC) scores, obtained from nine external measurements, from 177 juvenile lesser snow geese, banded or collected in southwest Louisiana in winters 2002–2004. Models used for interpretation are shown in bold (see text for details)

Models a	K b	AIC	ΔAIC	Wi	LogL
PC1: Overall body size					
**Sex + Habitat + Method**	**5**	**745.2**	**0.0**	**0.27**	**−367.6**
Sex + Habitat	4	745.3	0.1	0.26	−368.7
Sex + Method	4	745.4	0.2	0.24	−368.7
Sex	3	745.9	0.7	0.19	−370.0
Sex + Habitat + Method + Sampling date	6	749.8	4.6	0.03	−368.9
S + H + M + S. date + S. date × Habitat	7	751.8	6.6	0.01	−368.9
Intercept model (null model)	2	782.6	37.4	0.00	−389.3
PC2: Culmen length, bill nares, and gape length relative to skull and wing					
**Sampling date**	**3**	**502.1**	**0.0**	**0.73**	**−248.0**
S + H + M + S. date + S. date × Habitat	7	504.6	2.6	0.20	−245.3
Sampling date × Habitat c	5	506.8	4.8	0.07	−248.2
Intercept model (null model)	2	532.8	30.7	0.00	−264.4
PC3: Bill thickness					
** Sampling date**	**3**	**424.5**	**0.0**	**0.86**	**−209.3**
Sampling date × Habitat c	5	428.7	4.1	0.11	−209.2
Intercept model (null model)	2	435.6	11.1	0.00	−215.8

a Only models with some support (ΔAIC < 10; provided they had lower AIC than the respective intercept model) and all intercept models are presented.

b K = number of parameters as reported by AICcmodavg package in R.

c Model contains both main effects as well.

**Figure 6:**
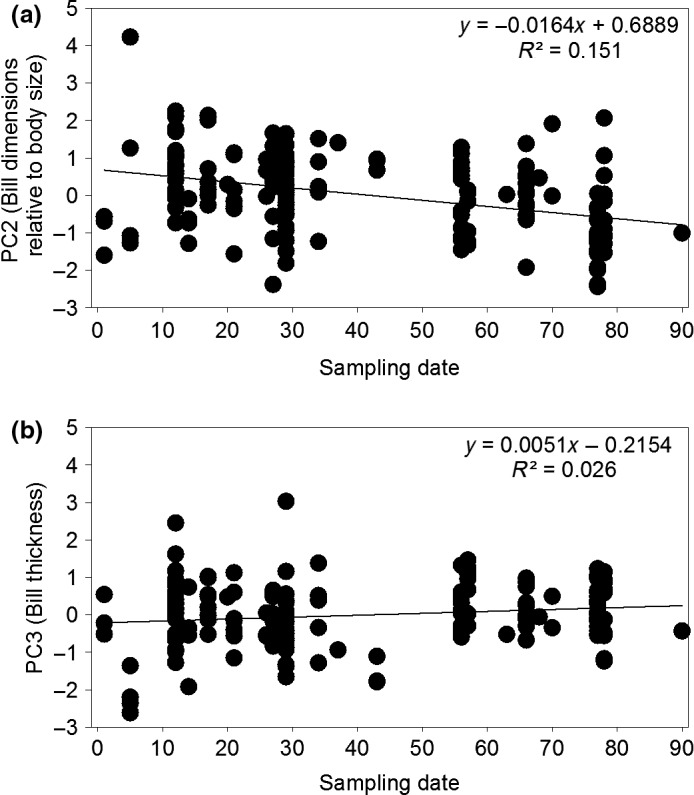
Sampling date and principal scores from external measurements of juvenile snow goose (*Chen caerulescens caerulescens*) specimens (*n* = 171) banded or collected in southwest Louisiana during winters 2001–2004. (a) the second principal score (PC2) which was interpreted as increased bill dimensions relative to body size with increased sampling date; (b) the third principal score (PC3) which was interpreted as increased bill thickness with increased sampling date

For PC2 (which opposed bill dimensions relative to the rest of the body), the top‐ranked model included only sampling date (Wi = 0.73), but other variables were not supported (Table 4). PC2 was inversely related (*R* ^2^ = .151) to sampling date (Figure 6a), which we interpret as bill dimensions (negative loadings) becoming smaller relative to wing length and skull dimensions (positive loadings) as winter progressed.

For PC3 (indicating bill thickness), the top‐ranked model included only sampling date (Wi = 0.86), but other variables were not supported (Table 4). PC3 was positively related (*R* ^2^ = .151) to sampling date (Figure 6b). There was no support for the exertion hypothesis as inferred from the external measurements, indicated by little support for the habitat × sampling date interaction for PC1 (ΔAIC ≥ 6.6), PC2 (ΔAIC ≥ 2.6), or PC3 (ΔAIC ≥ 4.1).

#### 3.1.4 Linear mixed models on PC scores for muscle measurements

**Figure 7:**
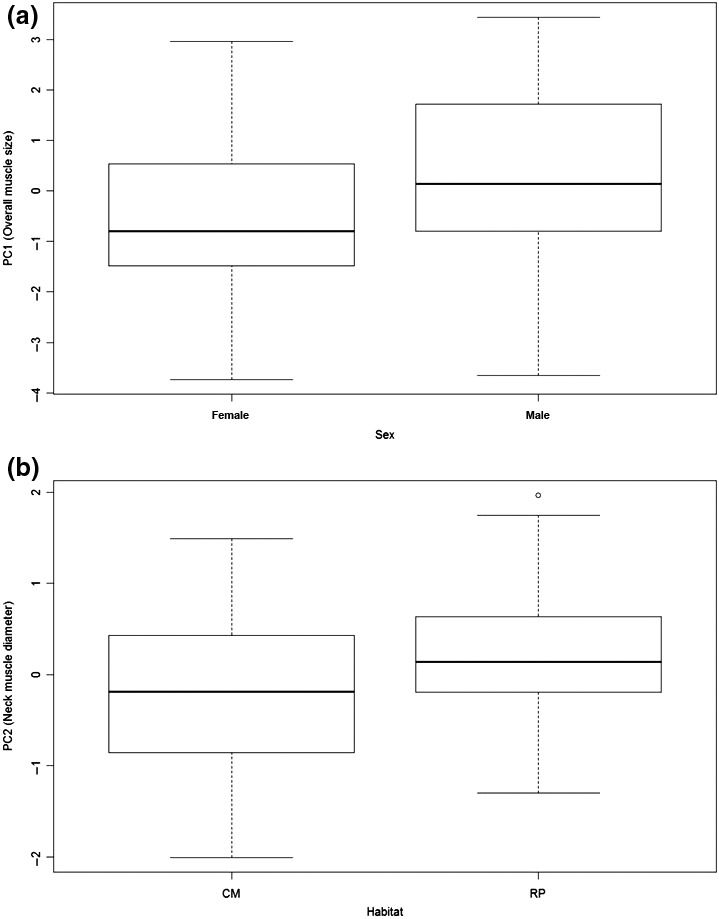
Tukey boxplots (the length of the box is the interquartile range, whiskers are drawn to the largest observations within 1.5 interquartile lengths from the top and bottom) of the differences in overall muscle size between the sexes (a) and neck muscle diameter between coastal marshes (CM) and rice‐prairies (RP) (b) based on muscle measurements of juvenile snow goose (*Chen caerulescens caerulescens*) specimens (*n* = 71) collected in southwest Louisiana during winters 2001–2004

For PC1 (which represented overall muscle size), the single‐effects model for sex (Wi = 0.35) was best supported, whereas there was no support for other models, with habitat a probable pretender variable given little change in LogL (0.7) between sex and sex + habitat (Table 5; Appendix S2 for full model selection tables). Sex and habitat had cumulative weights of 0.71 and 0.44, respectively, indicating that sex was the more important variable. On average, males had larger muscle measurements than did females (Figure 7a), similar to the sex differences in body size (Figure 5a).

**Table 5:** Linear mixed models testing effects of sex, habitat, sampling date, and habitat × sampling date on snow goose musculature. Dependent variable were each of four principal components (PC) scores, obtained from nine muscle measurements from 71 juvenile lesser snow geese collected in southwest Louisiana in winters 2002–2004. Models used for interpretation are shown in bold (see text for details)

Models a	K b	AIC	ΔAIC	Wi	LogL
PC1: Overall muscle size					
**Sex**	**3**	**280.9**	**0.0**	**0.35**	**−137.3**
Sex + Habitat	4	281.7	0.8	0.23	−136.6
Intercept model (null model)	2	282.4	1.5	0.17	−139.1
PC2: Neck muscle diameter					
**Habitat**	**3**	**181.6**	**0.0**	**0.59**	**−87.6**
Sex + Habitat	4	183.1	1.5	0.29	−87.2
PC3: Skull muscle diameter					
**Intercept model (null model)**	**2**	**175.1**	**0.0**	**0.51**	**−85.5**

a Only models with some support (ΔAIC < 10; provided they had lower AIC than the respective intercept model) and all intercept models are presented.

b K = number of parameters as reported by AICcmodavg package in R.

For PC2 (which represented neck muscle diameter), the single‐effects model for habitat (Wi = 0.59) was the most important, whereas there was no support for other models, with sex a probable pretender variable not causing much change in LogL (0.4) between habitat and sex + habitat (Table 5). Habitat and sex had cumulative weights of 0.89 and 0.39, respectively, indicating that habitat was the more important variable. On average, snow geese from coastal marshes had larger neck muscle diameter than did those from rice‐prairies, but those from coastal marshes also were more variable (Figure 7b).

For PC3, the intercept model was the best supported model (Wi = 0.51), indicating that none of our explanatory variables meaningfully explained any variation in PC3 (Table 5). Thus, we did not consider PC3 from muscle measurements further in this study. There was no support for the exertion hypothesis as inferred from the muscle measurements, indicated by little support for the habitat × sampling date interaction for PC1 (ΔAIC ≥ 5.0), PC2 (ΔAIC ≥ 8.0), and PC3 (ΔAIC ≥ 9.7).

### 3.2 The test of the introgressive hybridization hypothesis

Frequency distributions were analyzed for all nine external measurements, stratified by method (banded or collected), habitat (coastal marshes or rice‐prairies), and sex (female or male), that is, a total of 72 distributions. Student's *t*‐tests indicated that the median did not differ from the mean in 68 of 72 distributions (*p* < .05). After adjusting for false discovery rates (FDR) within each set of eight tests per each of the nine external measurements, the four distributions where mean and median differed were (1) head width of banded males in both habitats (*t* = 3.35, *p* = .002, FDR α = .00625; and *t* = 3.23, *p* = .004, FDR α = .0125, for coastal‐marsh and rice‐prairie males, respectively); and (2) head height of banded rice‐prairie females (*t* = 2.92, *p* = .009, FDR α = .0125) and collected specimens of coastal‐marsh females (*t* = −4.50, *p* = .0002, FDR α = .00625). Overall, the generally similar means and medians (which often differed by no more than ≤1.0 mm) indicated that there was little skewness in the data and, thus, no indication of hybridization and concomitantly no support for the introgressive hybridization hypothesis.

Visual inspection of the 72 frequency distributions (Appendix S3) revealed: (1) long tails toward the largest rice‐prairie males for culmen length, bill nares, and gape length; (2) that distributions for measurements from rice‐prairies seem platykurtic or “flattened” (and, thus, more variable) relative to those from coastal marshes for head length, head width, and head height; and (3) that there were no signs of bimodality detected in the data when they are stratified by method, sex, or habitat.

## 4 Discussion

We found that measurements of the head, bill, and muscles were similar for juvenile snow geese from rice‐prairies and coastal marshes, which indicate that the observed differences between the two observed morphs are unlikely to be the result of differences in their physical activities during winter foraging. Thus, our findings failed to support the exertion hypothesis. Our results also failed to support the introgressive hybridization hypothesis with respect to Ross's goose (Alisauskas, 1998), as there was a general lack of skewness and bimodality in the outer measurements and no differences between habitats where the skewness occurred. For the effects of such hybridization to be inferred from the morphological data, the distributions of bill sizes would be skewed to the left (with median differing from the mean) and would have had longer tails than those observed in this study.

Of the effects in our linear mixed models, sex generally was more important in explaining variation in both external and muscle measurements, which is in agreement with previous studies on adult snow geese in that males were larger (Alisauskas, 1998; Aubry et al., 2013 Cooch et al., 1996, 1997; Cooke et al., 1995; Jónsson, 2005). Habitat generally was not important in predicting our PC scores, except for neck muscle measurements (PC2 in muscle measurements), and the general lack of habitat effects contrasts with previous findings on external measurements in adult snow geese, where habitat explained variation in PC scores (Alisauskas, 1998; Jónsson, 2005). Method generally was relatively unimportant for explaining variation in external measurements. Bill dimensions (PC2 in external measurements) and bill thickness (PC3 in external measurements) changed only slightly with sampling date. Obviously, there is no reason to interpret the negative relationships between PC2 and sampling date as the bill is “becoming smaller.” Rather, the head but not the bill grew during our sampling period or that juveniles with different morphologies arrive to the study area at different dates. Furthermore, sampling date rarely was important in our analyses, indicating either slow growth during the winter months or large individual variation in the bill sizes (see Jónsson, 2005). Furthermore, this “shrinking” could be a result of larger birds migrating north earlier, leaving behind only smaller birds during late winter.

Both body size indices (PC1 in both datasets) differed by sex but not habitat, and overall body size did not change from November to February. PC1 represented body size and 53% and 60% of the overall variation in external measurements and muscle measurements, respectively. Thus, once body size had been accounted for, there seemed to be little room left for meaningful PC scores, which usually explain <10% of the variation in external measurements (Alisauskas, 1998; Jónsson, 2005; Jónsson et al., 2014; this study). PC2 represented all bill dimensions except bill thickness (PC3) and had a meaningful eigenvalue. Bill dimensions also comprised the PC2 in the study of Alisauskas (1998), whereas those were scattered over PC2–PC5 in the study of Jónsson et al. (2014), where PC2 also included head height and head length. Taken together, these studies show that once PC1 has accounted for body size, the next PC scores represent the bill or skull dimensions, which can behave quite independently of body size. Our findings here suggest that body size (PC1) does not grow appreciably in juvenile snow geese during winter, but some body parts grow fast relative to others (PC2), and bill thickness (PC3) increases and seemingly does so independently of habitat. Bill morphology seems less constrained when responding to selective pressures than other avian body parts, such as wings or legs (Benkman, 1993), and thus, it is not surprising that bill morphology in snow geese is independent of body size. Interrelationships of bill dimensions within PC scores differ between studies and probably also years within studies (Alisauskas, 1998).

### 4.1 Segregation of morphs by habitat

A banding study in winters 2001–2004 showed that movements of banded birds were frequent from marshes to rice‐prairies, and movement probabilities were independent of body size (Jónsson et al., 2014). However, movement probabilities depended on intervals (i.e., differed between sampling periods within the study period), which indicated that the snow geese responded to shifts in environmental conditions. Thus, coastal‐marsh snow geese and rice snow geese generally remain segregated, but events cause them to integrate during periods of high movements, which may occur as commonly as every 1–3 years apart. The distribution of bill sizes within a population can vary annually in response to changing environmental conditions (Grant & Grant, 2002), including those of snow geese (Alisauskas, 1998; Jónsson, 2005).

Juvenile snow geese are 4–8 months old during their stay in southwest Louisiana, assuming that eggs hatch in the beginning of July (Jónsson, Afton, & Alisauskas, 2007). Thus, the size of various body parts may be influenced by their activities during their first wintering season or perhaps their hatch dates (Cooke et al., 1995). As adults show segregation into habitats by body size, we would expect the same in juveniles, given that structural size has a genetic component. Bill size and body size also have a genetic component and are moderately to highly heritable in birds (Abzhanov, Protas, Grant, Grant, & Tabin, 2004; Francis & Guralnick, 2010; Husby, Hille, & Visser, 2011). Body size variation has an environmental component as well; for example, body size in several birds, including lesser snow geese, has declined in recent decades, possibly in response to climate change (Aubry et al., 2013; Cooch, Lank, Rockwell, & Cooke, 1991; Husby et al., 2011; Van Buskirk, Mulvihill, & Leberman, 2010), climatic variability, or primary productivity (Goodman, Lebuhn, Seavy, Gardali, & Bluso‐demers, 2012). Furthermore, there is annual variation in the morphological segregation into habitats which corresponds with variable movement probabilities between habitats between seasons (Alisauskas, 1998; Jónsson et al., 2014).

It remains unresolved why geese continue to use both agricultural and marsh habitats, despite higher energy intakes gained from agricultural foods. In a study of barnacle geese (*Branta leucopsis*) in the Dutch Wadden Sea, Eichhorn, Meijer, Oosterbeek, and Klaasen (2012) found that geese that foraged in intensively managed agricultural pasture maintained an adequate amino acid level in their diet, when compared to those feeding in natural salt marshes. In fact, food from pasture salt marsh and natural salt marshes were similar with respect to overall amino acid content and composition. However, we suggest that continued mixed strategy of using both agricultural and natural habitats among geese may be maintained by a variety of factors, such as disturbance levels, or because of other nutrient needs such as mineral requirements (Jónsson et al., 2014). Diet and nutritional value are not the only drivers of animal behavior in the Northern Hemisphere, and their relationships with genetics need to be considered for snow geese (Larsson et al., 1997, 1998).

There has been a shift toward smaller body size in snow geese in recent decades, among breeding birds in La Perouse Bay (Cooch, Lank, Rockwell, et al., 1991) and also in the midcontinent population (Alisauskas, 2002), which was concurrent with increased use of agricultural fields instead of natural wetlands (Alisauskas, 1998) and prolonged short‐stopping on the staging grounds (Jónsson & Afton, 2015). This shift in habitat use toward agricultural fields in migration areas to the north of Louisiana could have favored individuals with smaller bills, as these agricultural staging grounds require similar feeding methods as the rice‐prairies.

Migration strategies could differ between the smaller‐ and larger‐billed snow geese (rice‐prairie and coastal‐marsh morphotypes); that is, perhaps smaller‐billed geese stop over longer up north, in agricultural habitats, and the bigger‐billed snow geese move more quickly to the south, toward the Gulf Coast. Similarly, larger‐bodied individuals have higher fasting endurances and can carry proportionately larger reserves, and thus can be more flexible to make longer migration flights than can smaller individuals, given adequate stored reserves (lipids). Thus, there could be variability in when individuals arrive in southwest Louisiana in early winter, or when they leave to migrate north in late winter, possibly violating our assumption of no differential migrations in or out of the study area during our sampling period. However, we know of no empirical data which suggest that larger individuals systematically arrive or leave the wintering grounds early, or that smaller individuals systematically arrive or leave the wintering grounds later. For example, body size did not predict the probability of adult snow geese moving between rice‐prairies and coastal marshes (Jónsson et al., 2014).

### 4.2 Implications for the possible evolution of Ross's geese from snow geese

The ecological constraints of small bill size for geese are even more sharply evident in Ross's geese, which have relatively small bills and are adapted for grazing on grass (Alisauskas, 1998; Jónsson, 2005). Although our morphological data provided no evidence for hybridization, the available genetic evidence suggests a shared evolutionary history between the two species (Weckstein et al., 2002). Ross's geese are two‐thirds the size of lesser snow geese, with a shorter neck and a diminutive and rounded head (Jónsson et al., 2013). Their bill is “high at the base, tapers steeply to a rounded tip, has a slight arch in the tomium of maxilla and mandible, but lacks the prominent dark ‘grinning’ or ‘smile’ patch characteristic of greater and lesser snow geese” (Jónsson et al., 2013). This species apparently is unable to grub for subterranean food in coastal marshes (McWilliams and Raveling 1998, Jónsson & Afton, 2008) although it can feed on such plant parts that have been dislodged by snow geese (JEJ unpublished observation). Ross's geese may have evolved from a single snow goose population (Jónsson et al., 2013). It is possible that smaller individuals first specialized on aboveground vegetation, a path that could be currently entered by the smaller “rice‐prairie” phenotype of the lesser snow geese. “Coastal‐marsh” and “rice‐prairie” snow geese may comprise a polymorphic metapopulation and may represent the beginning of diverging evolutionary paths based on bill size in adaptation to different foraging techniques, that is, the very beginning of an eventual speciation event. We hypothesize that a similar ecological segregation (smaller‐sized individuals pursuing diets that required less muscular exertion during feeding, progressing further down the path of evolution) contributed to the evolution of Ross's geese.

## 5 Conclusions

Our results provided no support for the exertion hypothesis as an explanation of the observed morphological differences between rice‐prairie and coastal‐marsh juvenile snow geese, but are congruent with the observations documented for adult snow geese by Alisauskas (1998). With respect to possible differences in exercise between habitats, the definitive morphological responses may have been too subtle and difficult to detect with the measurements we used, especially when the skull is involved.

Growth of snow goose goslings is sensitive to variation in food supply (Cooch et al., 1996). At the breeding grounds, female goslings that grew up in habitats that were long degraded (by overgrazing from superabundant snow goose populations) had lower body masses and lower survival compared to those that grew up in newly colonized areas (Aubry et al., 2013). It would of interest to learn whether these observed differences at the breeding grounds correlate with the habitat segregation in southwest Louisiana, that is, whether the goslings from the overgrazed areas become rice‐prairie juveniles and the goslings from the newly colonized areas become coastal‐marsh juveniles. This could depend on the rate of the winter site‐faithfulness shown by their parents, or the probability of family breakup which would lead to the orphaned goslings choosing their respective wintering areas by themselves.

Two main questions remain to be studied. One question is whether and how far genetic differentiation has progressed in the snow geese (but see Humphries et al., 2009). The other question is whether the snow geese in the wintering grounds in southwest Louisiana represent a polymorphic population with individual snow geese selecting their winter feeding habitats according to their own physical state [see the habitat selection hypothesis of Alisauskas (1998)] or whether the wintering snow geese represent adjacent metapopulations that differ morphologically and behaviorally [see the phenotypic selection hypothesis of Alisauskas (1998)]. Muscular exertion during feeding may be a mechanism for habitat selection; that is, marsh diets are easily utilized by larger‐billed individuals but avoided by smaller‐billed individuals, whereas rice‐prairie diets are suitable for all individuals but require less muscular exertion and are thus preferred by smaller‐billed individuals. Answers to these questions will require longitudinal studies (repeated measurements over the course of the winter) of individually banded snow geese in both their breeding and wintering grounds and over generations. We did not have repeated measurements on individuals to assess growth, but assumed we were measuring the same population throughout winter. Our measurements should index individual growth if this assumption is reasonable. Such future studies will have clear implications for conservation and also contribute significantly to the clarification of the mechanisms of adaptive evolution and speciation.

## Conflict of Interest

None declared.
