# Supplementary material for: Do foraging methods in winter affect morphology during growth in juvenile snow geese?
Source: Ecol Evol. 2016 Oct 5;6(21):7656–70. doi: 10.1002/ece3.2481 (PMC6093151; doi:10.1002/ece3.2481)

Electronic appendix 1: Full model selection tables for PC scores from external measurements, summarized in Table 4.

| *PC1: Overall body size* |  |  |  |  |  |
| --- | --- | --- | --- | --- | --- |
| **Model** | **K** | **AIC** | **ΔAIC** | **Wi** | **LogLik** |
| Sex + Habitat + Method | 5 | 745.2 | 0.0 | 0.27 | -367.6 |
| Sex + Habitat | 4 | 745.3 | 0.1 | 0.26 | -368.7 |
| Sex + Method | 4 | 745.4 | 0.2 | 0.24 | -368.7 |
| Sex | 3 | 745.9 | 0.7 | 0.19 | -370.0 |
| Sex + Habitat + Method + Sampling date | 6 | 749.8 | 4.6 | 0.03 | -368.9 |
| S + H + M + S. date + S. date*Habitat | 7 | 751.8 | 6.6 | 0.01 | -368.9 |
| Method | 3 | 782.4 | 37.2 | 0.00 | -388.2 |
| Intercept model (null model) | 2 | 782.6 | 37.4 | 0.00 | -389.3 |
| Sampling date | 3 | 783.1 | 37.9 | 0.00 | -388.6 |
| Habitat | 3 | 783.5 | 38.3 | 0.00 | -388.8 |
| Habitat + Method | 4 | 783.6 | 38.4 | 0.00 | -387.8 |
| Sampling date*Habitat | 5 | 787.1 | 41.9 | 0.00 | -388.4 |
|  |  |  |  |  |  |
| *PC2: Culmen length, bill nares and gape length relative to skull and wing* | | | |  |  |
| **Model** | **K** | **AIC** | **ΔAIC** | **Wi** | **LogLik** |
| Sampling date | 3 | 502.1 | 0.0 | 0.73 | -248.0 |
| S + H + M + S. date + S. date*Habitat | 7 | 504.6 | 2.6 | 0.20 | -245.3 |
| Sampling date*Habitat | 5 | 506.8 | 4.8 | 0.07 | -248.2 |
| Habitat + Method | 4 | 518.5 | 16.5 | 0.00 | -255.3 |
| Sex + Habitat + Method | 5 | 519.1 | 17.0 | 0.00 | -254.5 |
| Habitat | 3 | 524.6 | 22.5 | 0.00 | -259.3 |
| Sex + Habitat | 4 | 525.3 | 23.2 | 0.00 | -258.7 |
| Method | 3 | 525.6 | 23.5 | 0.00 | -259.8 |
| Sex + Method | 4 | 525.7 | 23.6 | 0.00 | -258.8 |
| Intercept model (null model) | 2 | 532.8 | 30.7 | 0.00 | -264.4 |
| Sex | 3 | 533.0 | 31.0 | 0.00 | -263.5 |
| Sex + Habitat + Method + Sampling date | 6 | 582.9 | 80.8 | 0.00 | -245.4 |

Electronic appendix continued next page.

Electronic appendix 1 continued.

| *PC3: Bill thickness* |  |  |  |  |  |
| --- | --- | --- | --- | --- | --- |
| **Model** | **K** | **AIC** | **ΔAIC** | **Wi** | **LogLik** |
| Sampling date | 3 | 424.5 | 0.0 | 0.86 | -209.3 |
| Sampling date*Habitat | 5 | 428.7 | 4.1 | 0.11 | -209.2 |
| Sex + Habitat + Method + Sampling date | 6 | 434.8 | 10.2 | 0.01 | -211.4 |
| Habitat | 3 | 435.1 | 10.6 | 0.00 | -214.6 |
| Intercept model (null model) | 2 | 435.6 | 11.1 | 0.00 | -215.8 |
| Sex + Habitat | 4 | 436.3 | 11.8 | 0.00 | -214.2 |
| S + H + M + S. date + S. date*Habitat | 7 | 436.3 | 11.8 | 0.00 | -211.2 |
| Habitat + Method | 4 | 436.5 | 12.0 | 0.00 | -214.3 |
| Sex | 3 | 436.6 | 12.0 | 0.00 | -215.3 |
| Method | 3 | 436.8 | 12.3 | 0.00 | -215.4 |
| Sex + Habitat + Method | 5 | 437.7 | 13.1 | 0.00 | -213.8 |
| Sex + Method | 4 | 437.7 | 13.2 | 0.00 | -214.9 |

Electronic appendix 2: Full model selection tables for PC scores from muscle measurements, summarized in Table 5.

| *PC1: Overall muscle size* |  |  |  |  |  |
| --- | --- | --- | --- | --- | --- |
| **Model** | **K** | **AIC** | **ΔAIC** | **Wi** | **LogL** |
| Sex | 3 | 280.9 | 0.0 | 0.35 | -137.3 |
| Sex + Habitat | 4 | 281.7 | 0.8 | 0.23 | -136.6 |
| Intercept model (null model) | 2 | 282.4 | 1.5 | 0.17 | -139.1 |
| Sex + Habitat + Sampling date | 5 | 283.6 | 2.7 | 0.09 | -136.3 |
| Habitat | 3 | 283.7 | 2.8 | 0.09 | -138.7 |
| Sampling date | 3 | 285.5 | 4.6 | 0.03 | -139.6 |
| S + H + S. date + S. date*Habitat | 6 | 285.9 | 5.0 | 0.03 | -136.3 |
| Sampling date*habitat | 5 | 289.2 | 8.3 | 0.01 | -139.1 |
|  |  |  |  |  |  |
| *PC2: Neck muscle diameter* |  |  |  |  |  |
| **Model** | **K** | **AIC** | **ΔAIC** | **Wi** | **LogL** |
| Habitat | 3 | 181.6 | 0.0 | 0.59 | -87.6 |
| Sex + Habitat | 4 | 183.1 | 1.5 | 0.29 | -87.2 |
| Intercept model (null model) | 2 | 186.1 | 4.6 | 0.06 | -91.0 |
| Sex | 3 | 188.1 | 6.5 | 0.02 | -90.8 |
| Sampling date | 3 | 189.0 | 7.5 | 0.01 | -91.3 |
| Sampling date*habitat | 5 | 189.6 | 8.0 | 0.01 | -89.3 |
| Sex + Habitat + Sampling date | 5 | 190.1 | 8.5 | 0.01 | -89.6 |
| S + H + S. date + S. date*Habitat | 6 | 192.5 | 10.9 | 0.00 | -89.6 |

Electronic appendix continued next page.

Electronic appendix 2 continued.

| *PC3: Skull muscle diameter* |  |  |  |  |  |
| --- | --- | --- | --- | --- | --- |
| **Model** | **K** | **AIC** | **ΔAIC** | **Wi** | **LogL** |
| Intercept model (null model) | 2 | 175.1 | 0.0 | 0.51 | -85.5 |
| Habitat | 3 | 177.2 | 2.0 | 0.18 | -85.4 |
| Sex | 3 | 177.3 | 2.1 | 0.17 | -85.5 |
| Sampling date | 3 | 179.1 | 4.0 | 0.07 | -86.4 |
| Sex + Habitat | 4 | 179.4 | 4.2 | 0.06 | -85.4 |
| Sampling date*habitat | 5 | 184.8 | 9.7 | 0.00 | -86.9 |
| Sex + Habitat + Sampling date | 5 | 186.1 | 11.0 | 0.00 | -87.6 |
| S + H + S. date + S. date*Habitat | 6 | 188.5 | 13.4 | 0.00 | -87.6 |

Electronic appendix 3. Frequency distributions were analyzed of all nine external measurements, stratified by method (banded or collected), habitat (coastal marshes or rice-prairies), and sex (female or male), i.e. a total of 72 distributions (Electronic appendix 3). Student’s t-test indicated that the median did not differ from the mean in 68 of 72 distributions. Each of nine variables is shown for each sex within each habitat, stratified by method (banded or collected).


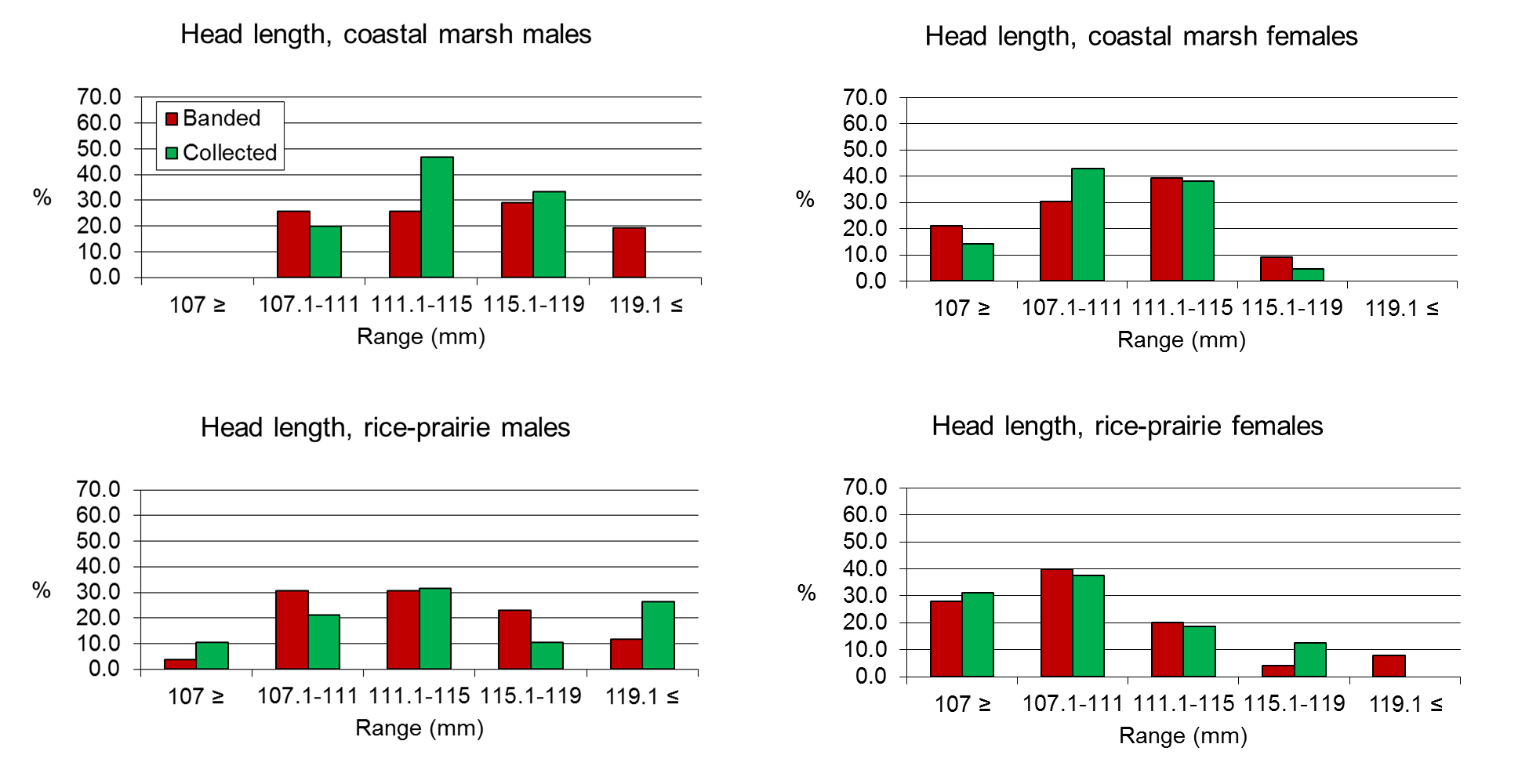


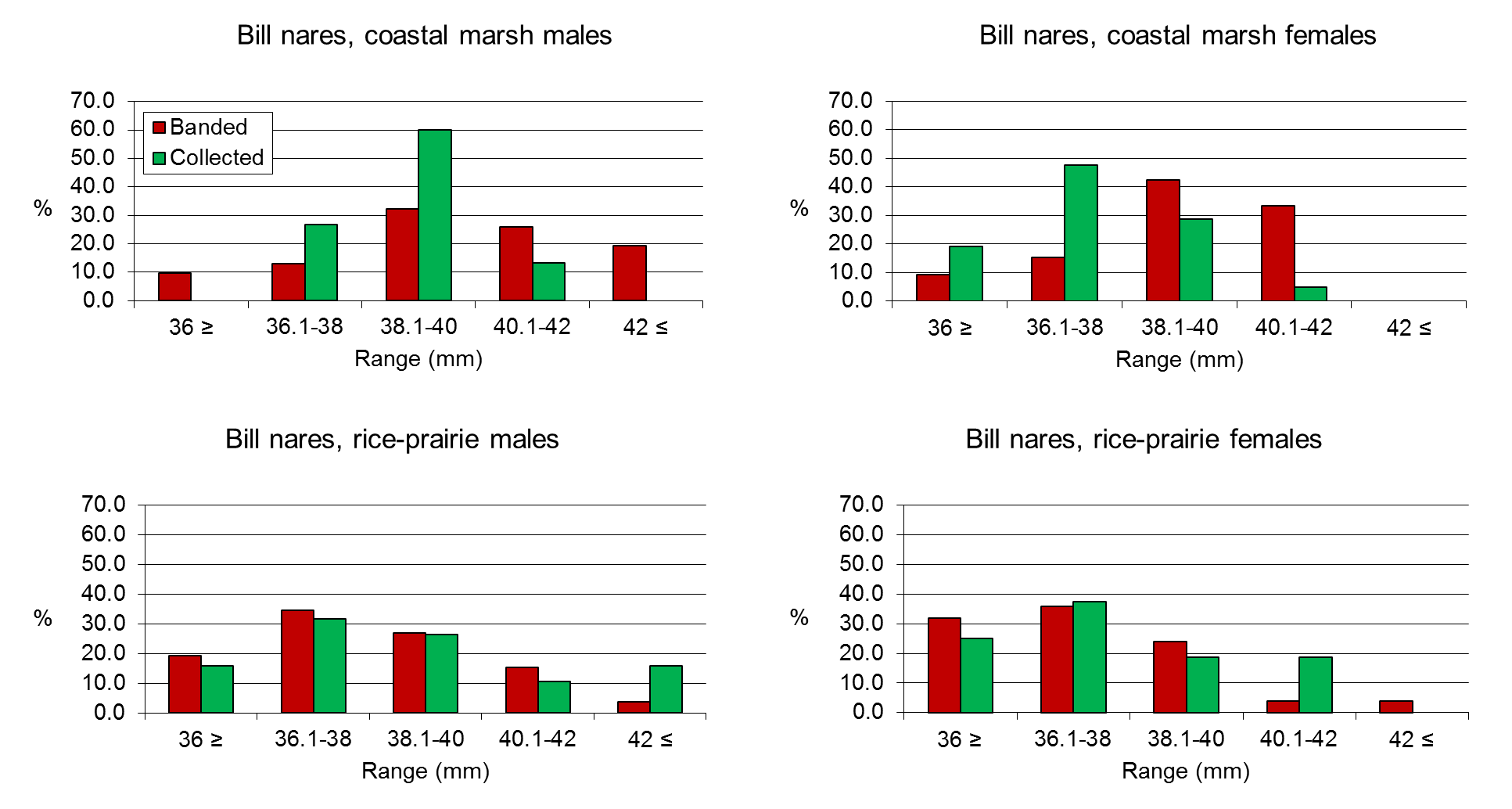


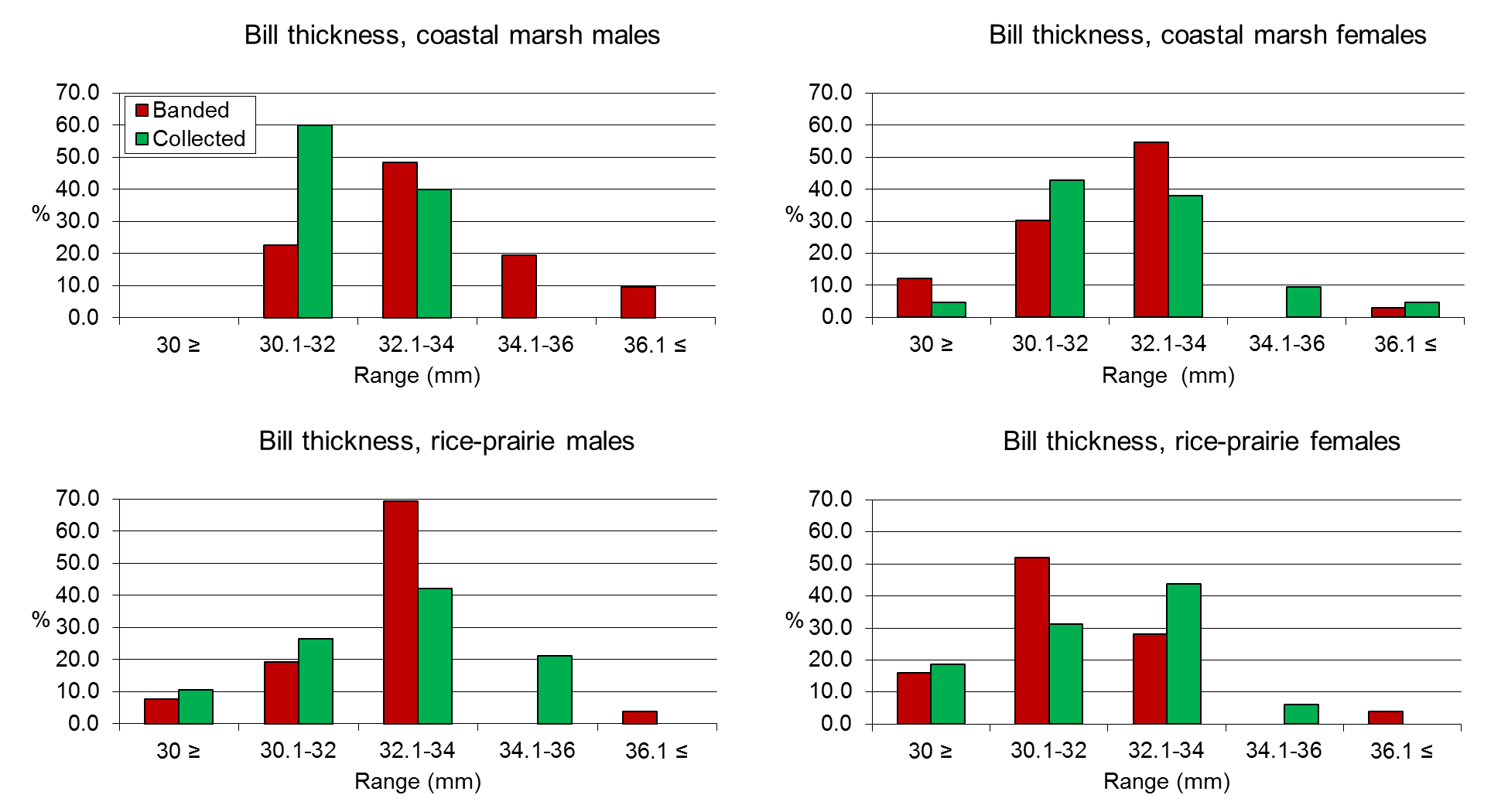


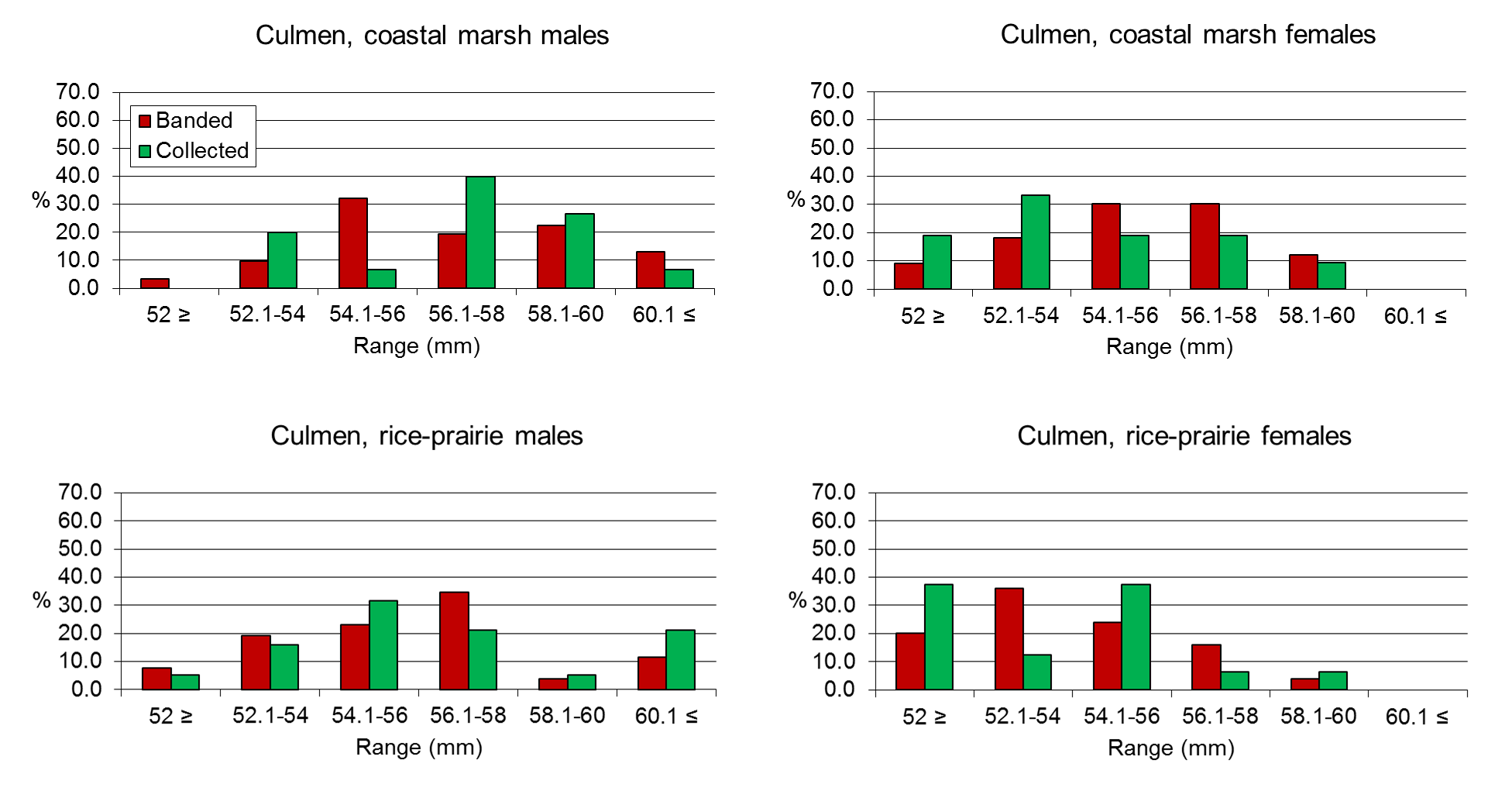


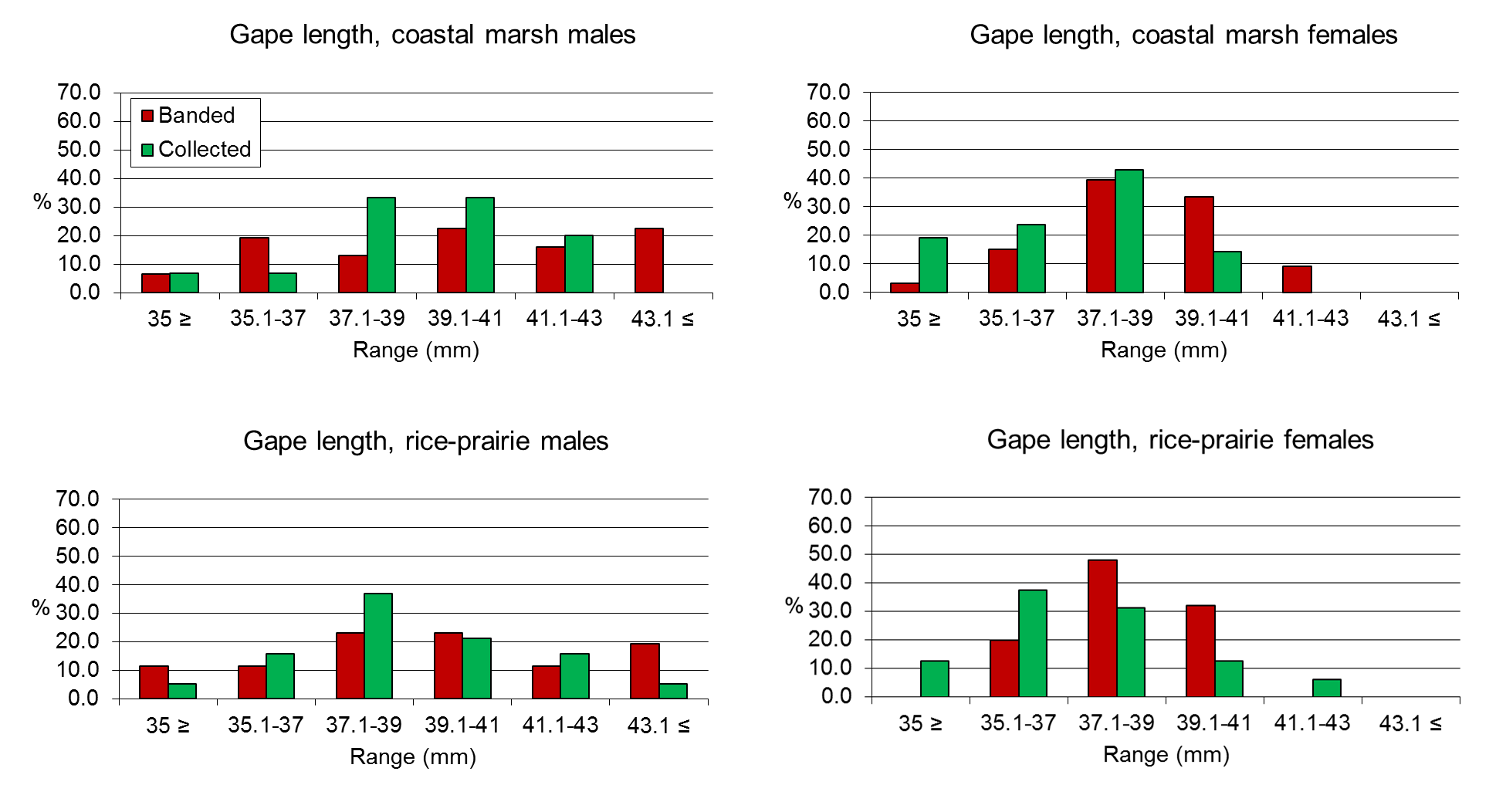


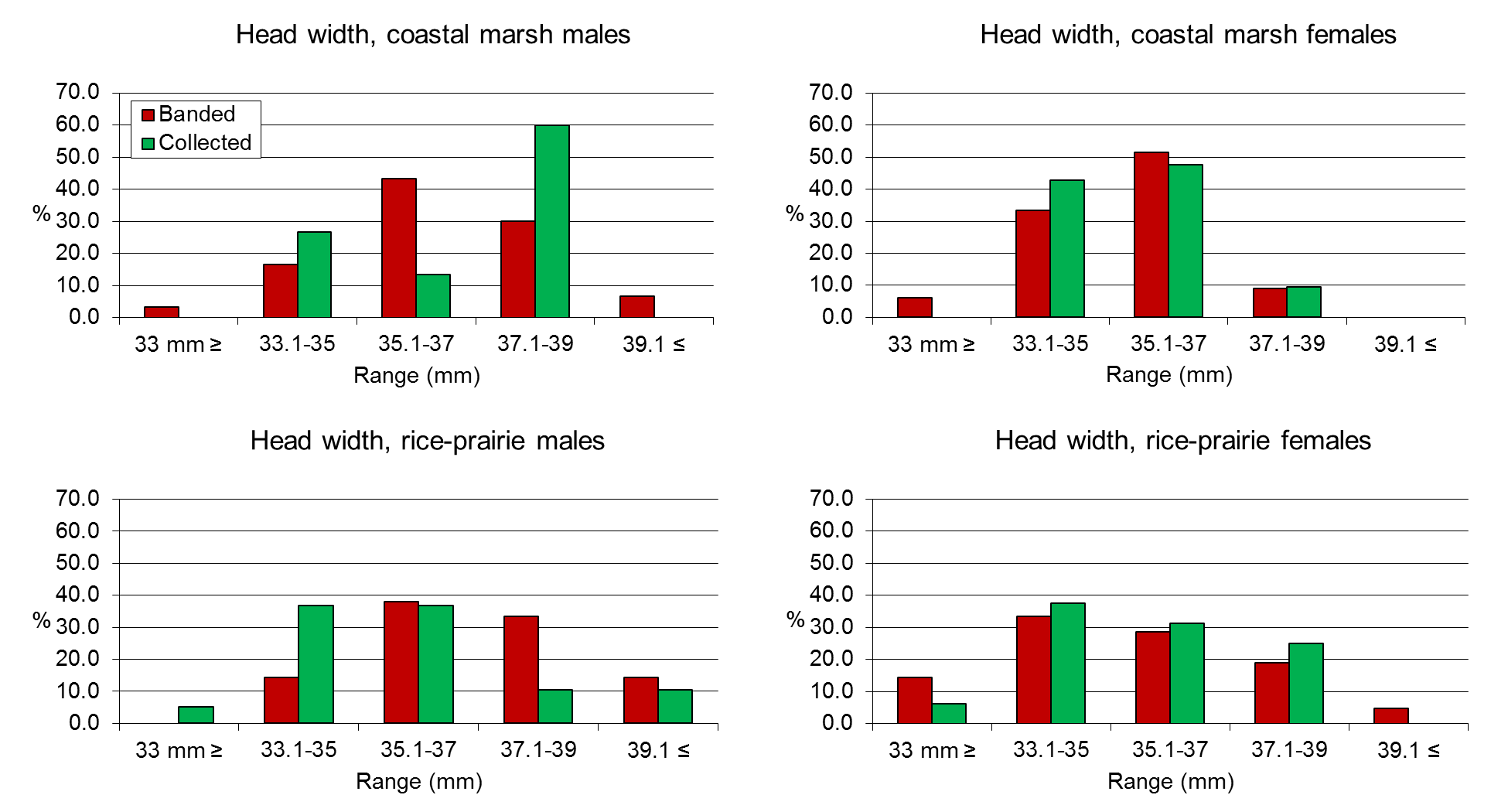


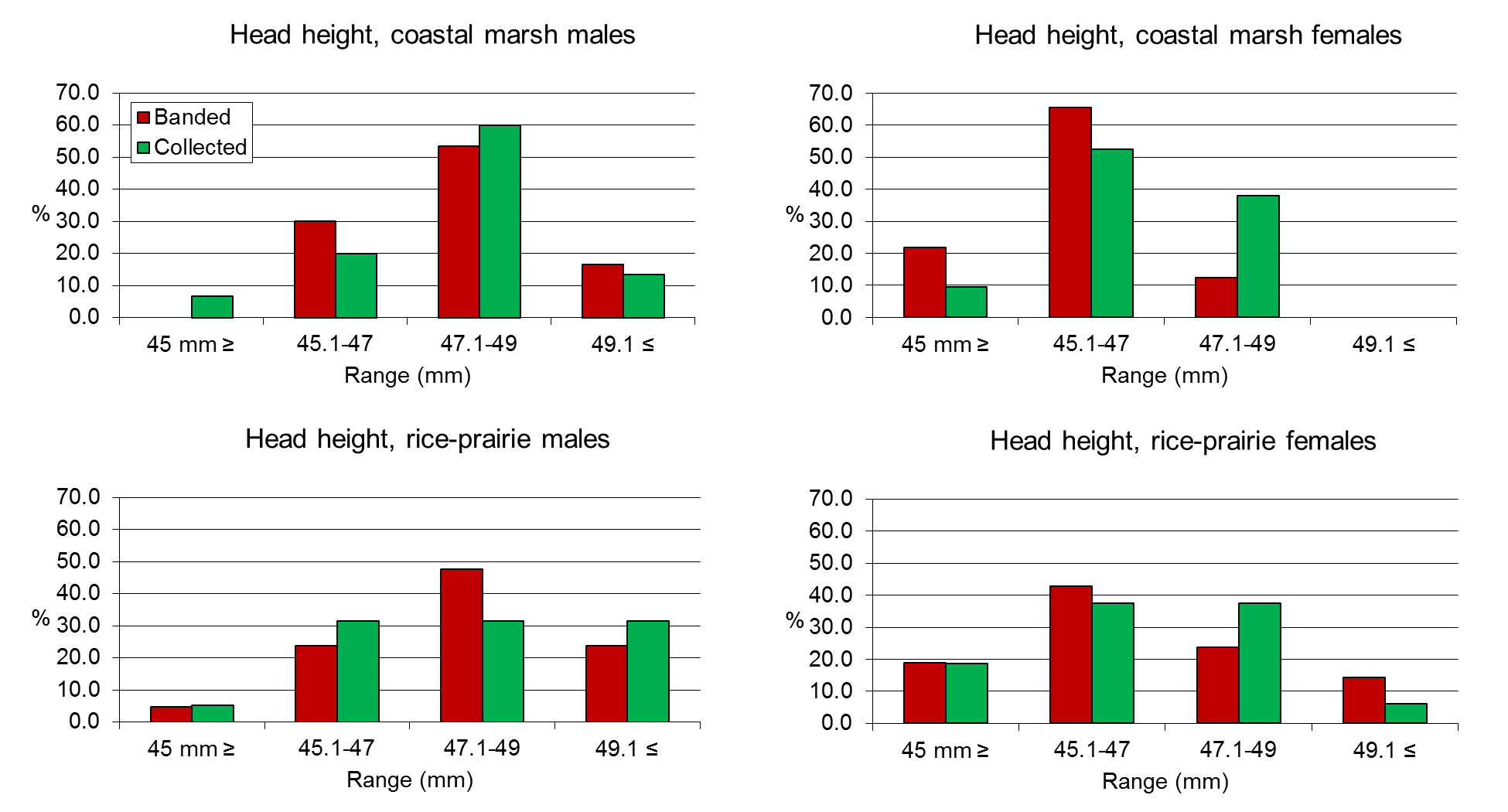


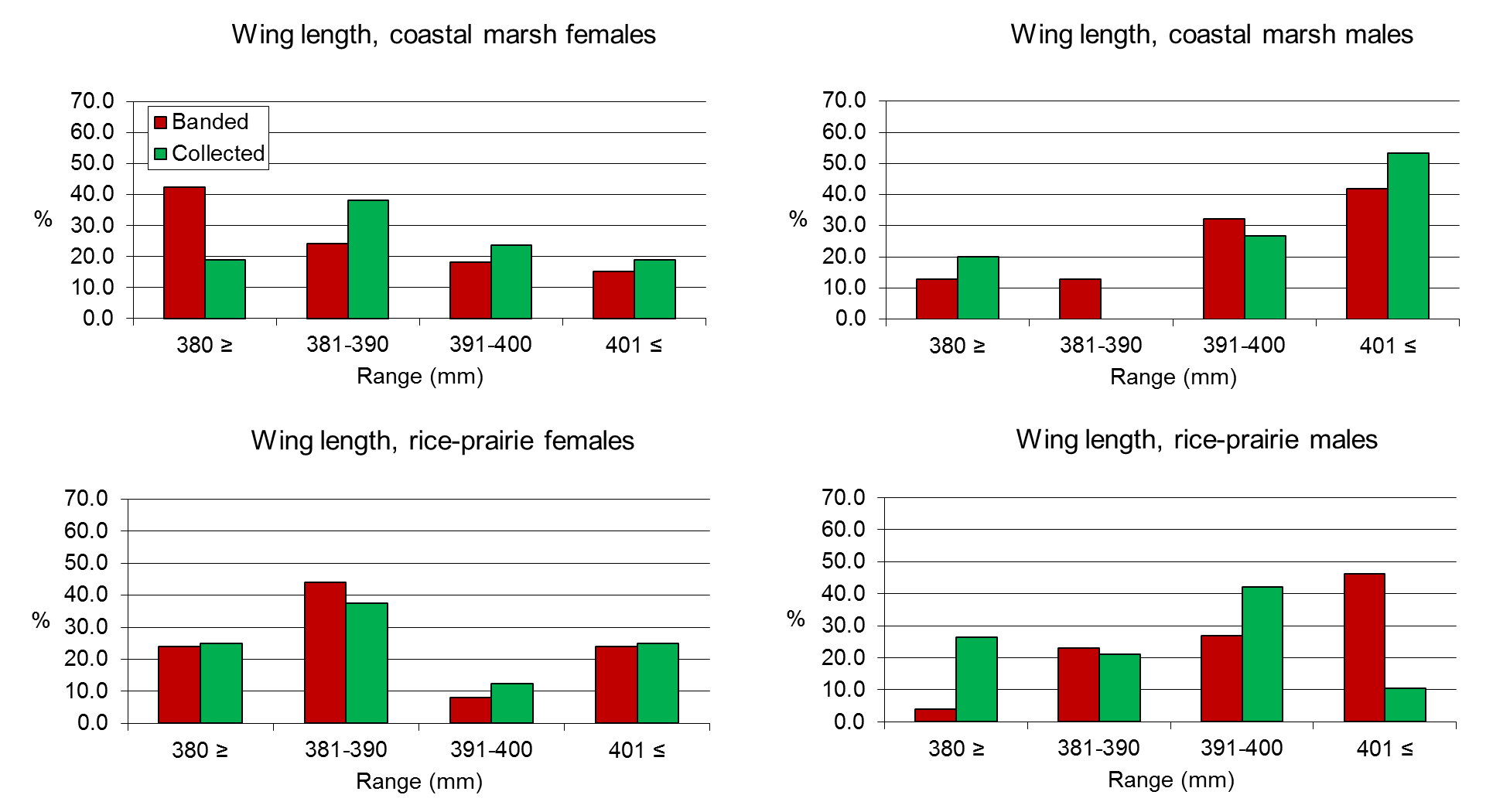


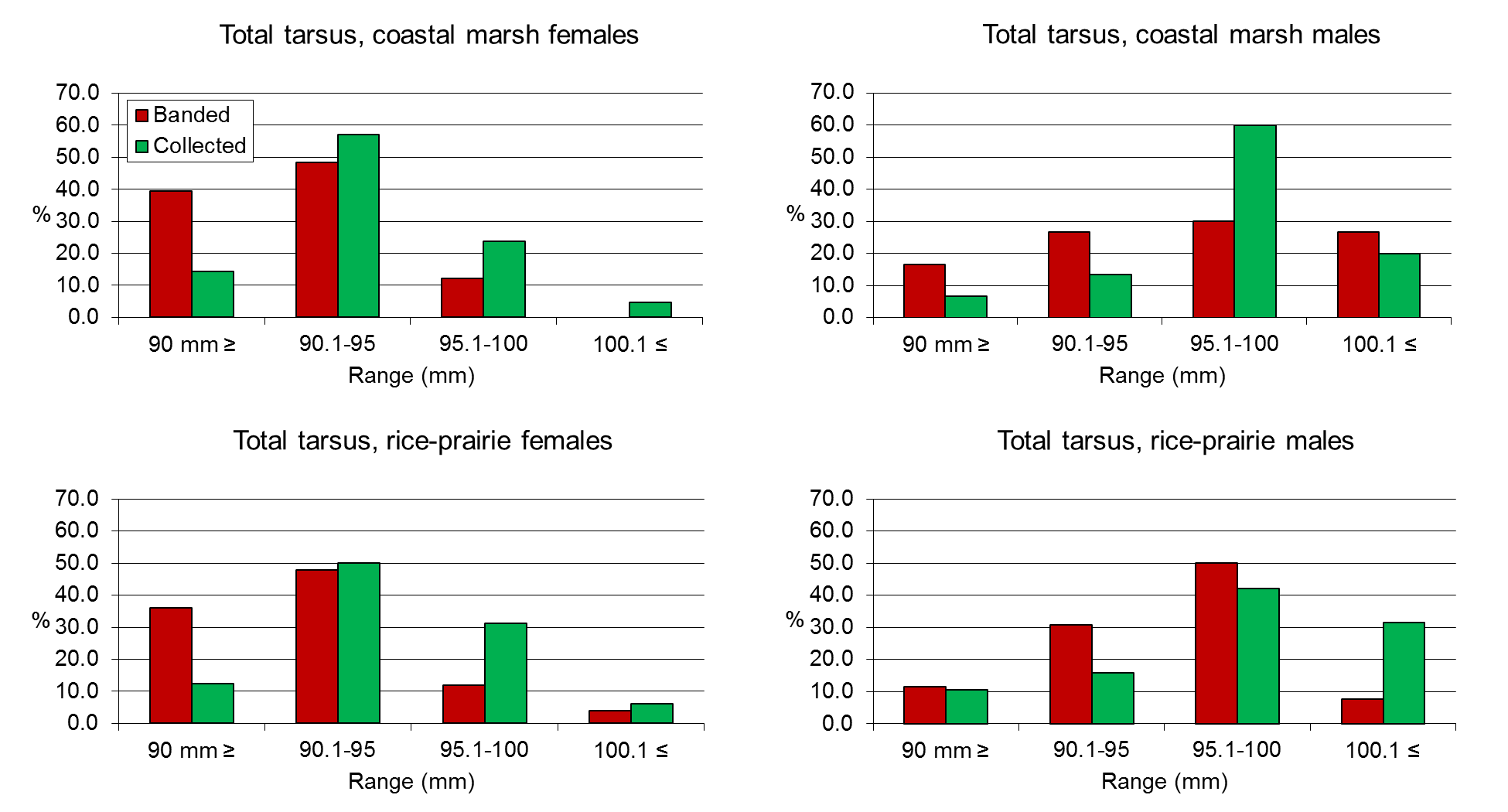

Supplement: Supplementary file 1 [file ECE3-6-7656-s001.docx]
